# Identification of Potential Inhibitors of *Mycobacterium tuberculosis* Amidases: An Integrated In Silico and Experimental Study

**DOI:** 10.1021/acsomega.4c07964

**Published:** 2024-11-06

**Authors:** Maciel Rosas-Cruz, Abraham Madariaga Mazón, Carlos D. García-Mejía, Eduardo Hernández-Vázquez, Homero Gómez-Velasco, Eva Jiménez-Faraco, Roberto Sealtiel Farías-Gaytán, Juan A. Hermoso, Siseth Martínez-Caballero

**Affiliations:** † Instituto de Química, Universidad Nacional Autonóma de México, A. Universidad 3000, Ciudad Universitaria, CP 04510 Ciudad de México, Mexico; ‡ Unidad Mérida del Instituto de Química, Universidad Nacional Autónoma de México, Km. 5.5 Carr. Sierra Papacal-Chuburna Pto. Sierra Papacal, CP 97302 Yucatán, Mexico; § Department of Crystallography and structural Biology, Instituto de Química-Física “Blas Cabrera”, Consejo Superior de Investigaciones Científicas, E-28006 Madrid, Spain

## Abstract

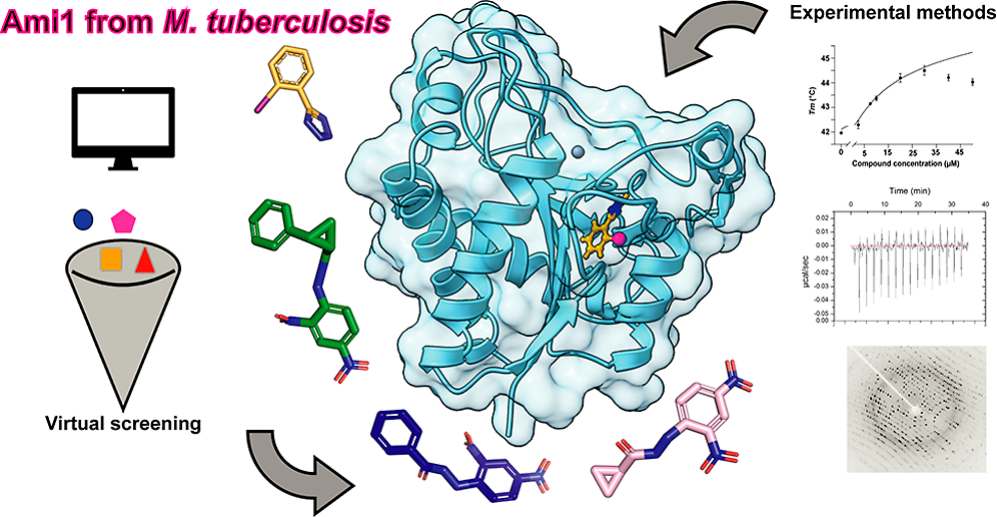

Virtual screening is a crucial tool in early stage drug discovery for identifying potential hit candidates. Here, we present an integrated approach that combines theoretical and experimental techniques to identify, for the first time, inhibitors of amidases (Ami1–Ami4) from *Mycobacterium tuberculosis*. Through computational methods, we proposed a set of potential inhibitors, which were subsequently evaluated experimentally using differential scanning fluorimetry. This led to the identification of two promising hits: a carbohydrazide core (hit **1**) and a tetrazole core (hit **5**). We further developed a small collection of compounds derived from hit **1**, which demonstrated improved affinity for Ami1. Additionally, we determined the crystallographic structure of the Ami1–hit **5** complex at a resolution of 1.45 Å, providing molecular-level insights into the interaction of this compound within the catalytic site. The findings of this study contribute to the advancement of drug discovery against tuberculosis and propose new targets for therapeutic development.

## Introduction

Antimicrobial Resistance (AMR) has become a serious global health problem. Since the World Health Organization (WHO) published its first report on the global status of Antibacterial Resistance (ABR) in 2014,^1^ updates to a priority list of bacterial pathogens of public health importance for research and development (R&D) have been periodically announced. Recently, the rifampicin-resistant (RR) pathogen *Mycobacterium tuberculosis* was included by the first time in the critical group of this list, alongside some Gram-negative bacteria.^2^

Although tuberculosis (TB) is a preventable and usually curable disease, it was estimated that in 2022, 1.30 million deaths worldwide were caused by this infection, making TB the second leading cause of death from a single infectious agent, after COVID-19.^3^ The increase in multidrug-resistant strains is also a matter of concern. According to the WHO, globally, 3.6% of new TB cases and 20.2% of previously treated cases are estimated to have multidrug-resistant TB (MDR-TB).^2^ Treatment regimens for MDR-TB are more expensive and toxic than those used for drug-susceptible TB (DS-TB), highlighting the need for new therapeutic strategies that combine the design of new antibiotics and the identification of new targets.

The mycobacterial cell wall is composed of three main components: peptidoglycan (PG), mycolic acid (MA), and arabinogalactan (AG). It has been reported that this cell wall plays a key role in intrinsic antibiotic resistance and virulence. PG is a macromolecule unique to bacteria that provides shape, rigidity, and integrity, allowing the cell to withstand turgor pressure and preventing lysis.^4^ Additionally, it is intimately involved in cell growth and division. These characteristics make it an attractive target for the development of new antibiotics. Therefore, studying the proteins involved in its synthesis and remodeling is a promising approach to identifying new targets for drug discovery.

PG is composed of a linear chain of alternating units of *N*-acetylglucosamine (NAG) and *N*-acetylmuramic acid (NAM) linked by β (1–4) bonds and cross-linked by short peptides.^5^ Although the core structure of PG is highly conserved, chemical modifications can be present in this macromolecule in some mycobacterial species, such as *N*-glycolylation of NAM,^6,7^ amidation in the peptide stems, the replacement of L-Ala by Gly in position 1 of the peptide stem, and the additional Gly residue bound to meso-diaminopimelic acid (mDAP).^8^

The regulation of PG synthesis and hydrolysis is crucial during bacterial growth, cell division, and cell separation.^9^ Generally, PG hydrolases can be classified according to their substrates as glycosidases and peptidases, which together perform the complete hydrolysis of the PG polymer into soluble fragments.^10^ Amidases, also known as *N*-acetylmuramyl-l-alanine hydrolases, break the bond between the L-Ala and NAM, separating the peptide stem from the linear glycan chain. These enzymes have been associated with PG degradation and turnover, cell separation, antibiotic resistance, and spore formation.^10,11^

Gene analysis of *M. tuberculosis* revealed the presence of four amidase homologues: Ami1 (Rv3717), Ami2/CwlM (Rv3915), Ami3 (Rv3811), and Ami4 (Rv3594).^12^ However, the functions of Ami3 and Ami4 remain unknown. Although hydrolytic activity has been demonstrated for Ami2/CwlM,^13^ its essential function is not as a hydrolase. Instead, it plays a principal role through its phosphorylation by PknB, regulating the biosynthesis of peptidoglycan precursors and their transport across the cytoplasmic membrane.^14,15^ The crystal structure of Ami1 has been solved, and its PG hydrolase activity has been demonstrated in vitro.^16,17^ Additionally, a study proposed that the absence or inactivation of this hydrolase in *M. tuberculosis* leads to cell wall alterations, increasing permeability and susceptibility to the frontline TB drug rifampicin. Furthermore, Ami1 is specifically required in *M. tuberculosis* during chronic mouse infection, as its amidase activity contributes to the pathogens ability to persist within the host.^18^

Strains of *Mycobacterium smegmatis* with individual and multiple deletions of Ami1, Ami3, and Ami4 exhibited normal growth and morphology.^19^ Similarly, deletions of Ami1 or Ami4 in *M. tuberculosis* suggest that these amidases are not essential for mycobacterial growth within the host.^20^ Both studies imply that the amidases of *Mycobacterium* may have redundant functions, as has been reported in other bacteria, such as *Escherichia coli*.^11,21,22^ In this study, we combined in silico and experimental approaches to identify a subset of molecules that showed good interaction with the catalytic domain of the four amidases. The experimental validation was conducted with Ami1, and a crystallographic complex was obtained at a resolution of 1.45 Å, allowing us to observe the molecular-level interaction of one of these compounds in the catalytic site.

## Results and Discussion

Information on the binding of small molecules to amidases is limited, and few studies have identified molecules that may interact with these PG hydrolases. In this work, we conducted a search for molecules based on two criteria: chemical cores with structural similarity to the substrate, and a search based on three previously reported inhibitors of Ami1_Mab_ from *Mycobacterium abscessus*(23) (Figure S1). These previously identified inhibitors were used as references to generate a molecular database aimed at identifying molecules that could inhibit to the amidase homologues (Ami1–Ami4) from *M. tuberculosis*. The generated molecular database comprised 35 molecules, with 10 showing similarity to reference inhibitor I, 19 similar to inhibitor II, and 6 similar to inhibitor III (Table S1). This database was then prepared for molecular docking with each of the amidase structures.

Prior to the molecular docking study, the binding sites predicted by the PocketFinder search algorithm^24^ were subjected to thorough analysis. For Ami1, the docking pocket was defined by the following residues: His35, Thr62, Glu70, Val06, His125, Ala126, Asp127, Arg183, Leu186, Ala187, Leu198, and Glu200. In contrast, the optimal pharmacophoric binding site for Ami2 included the residues Arg5, Leu70, Asp251, Arg270, Glu272, Trp325, Gln337, and Asp339. For Ami3, the identified interaction site comprised His227, Trp257, Tyr262, Phe287, His288, Thr289, Gly290, Tyr346, Thr347, His363, Gly367, and Asn368. Lastly, the binding site for Ami4 was characterized by the residues Gln166, Gly91, Asn104, Leu64, Trp88, His89, and Lys168 (Figure S2).

All the predicted binding sites include a region that involves the catalytic cavity of each amidase. A study by Prigozhin et al. identified residues His35, His125, and Glu70 as responsible of Zn^2+^ coordination, with Glu200 serving as the catalytic residue for Ami1.^16^ In the case of Ami2, which belongs to the Pfam Amidase_3 zinc-dependent family along with Ami1, the corresponding residues are Arg199, Arg270, and Glu217, with Asp339 as the catalytic residue (Figure S3A). Despite the replacement of His residues with Arg in the Zn^2+^ coordination triad and the substitution of Asp with Glu as a catalytic amino acid, muralytic activity has been experimentally confirmed for this protein.^13^

Although the catalytic activities of Ami3 and Ami4 have not been studied, our analysis revealed a 28.65% sequence identity between the amidase domains of Ami3 and BbtPGRP3 from *Branchiostoma belcheri tsingtauense*. Structural superposition of the crystallographic structure of the amidase domain of BbtPGRP3 (PDB: 4Z8I)^25^ with the AlphaFold^26,27^ model of Ami3 (AF-Q79F96) suggests that the residues His227, Tyr 262, His 363 and Cys371 in Ami3 could be responsible for catalytic activity (Figure S3B). Additionally, Ami4 showed a 20.99% sequence identity with AmpD from *Citrobacter freundii* (PDB: 2Y2C).^28^ A structural superposition between these proteins suggests that the residues His45, Glu111, His158 and Asp170 in Ami4 (AF-I6Y3Z2) may be important for catalysis (Figure S3C).

**Figure 1:**
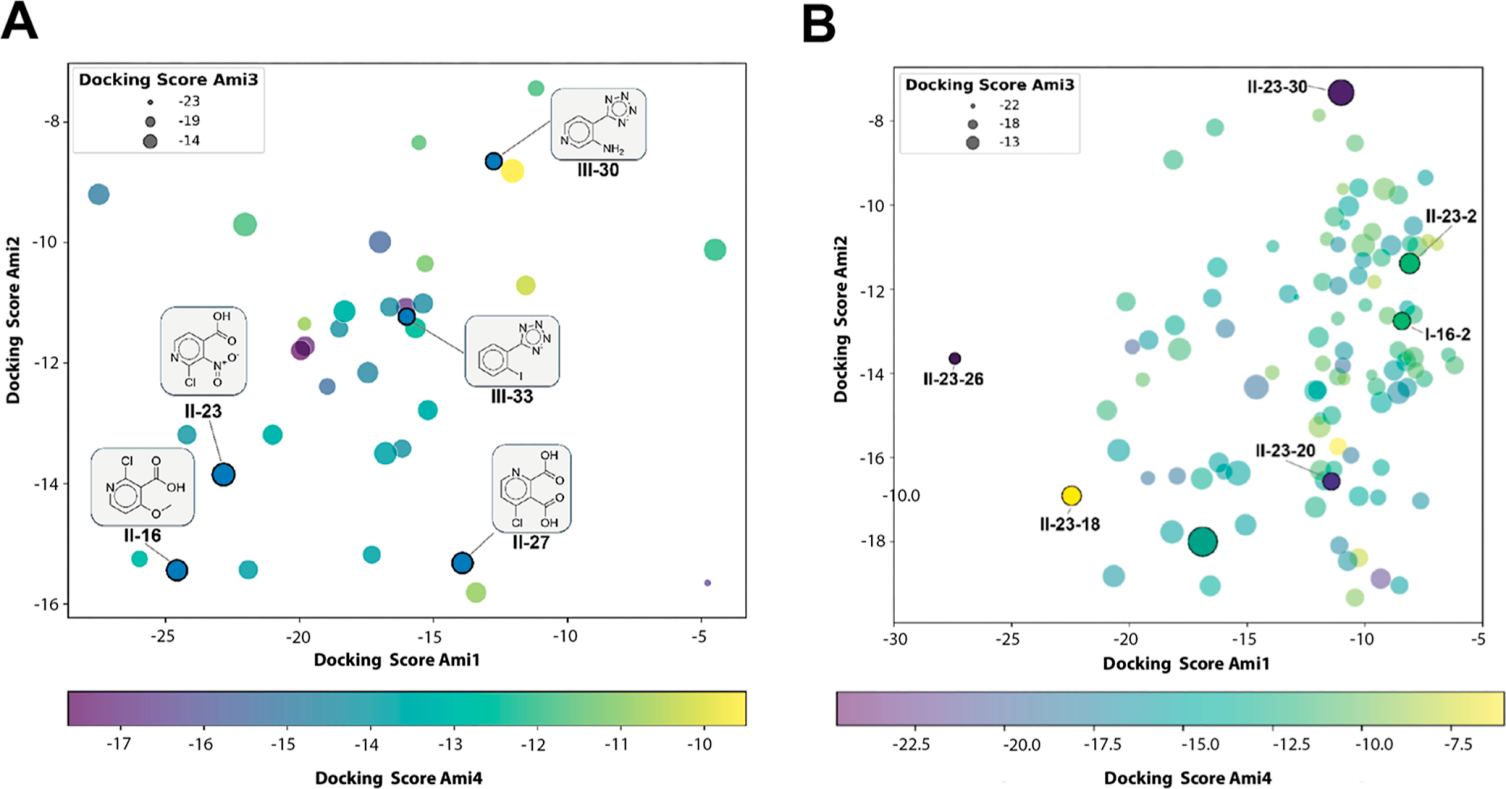
(A) Scatter plot displaying the docking scores for 35 molecules across four different amidases. (B) Scatter plot representing the docking scores for the expanded database of 108 molecules against the four amidases. In both plots, the docking scores for Ami1are plotted on the *X*-axis, while the scores for Ami2 are plotted on the *Y*-axis. Each point represents a molecule, with its position determined by its docking scores against Ami1 and Ami2. The size of each marker reflects the docking score for Ami3, with smaller markers indicating better docking scores (more negative values). The color of each marker represents the docking score for Ami4, using the color gradient, where darker colors correspond to better docking scores (more negative values). Selected molecules are highlighted full-colored markers and are identified by their ID in the molecular database.

After identifying the binding sites, molecular docking was performed. The results are summarized in Table S2, where negative values indicate a theoretically favorable interaction. Figure 1A provides an overview of the molecular docking results: the docking score for Ami1 is represented on the “*X*” axis, the docking score for Ami2 on the “*Y*” axis, the docking score for Ami3 by marker size, and the docking score for Ami4 by marker color code. Molecules located in the lower left quadrant, with darker and smaller markers, indicate significant interaction with all four amidases.

As depicted in Figure 1A, five molecules were selected as potential amidase inhibitors. Among these, II-16 and II-23 are particularly interesting due to their strong docking scores for Ami1 and Ami2 as well as reasonable scores for Ami3 and Ami4. Notably, both molecules are similar to reference inhibitor II.

To expand the chemical space of potential amidase inhibitors, a second similarity search was conducted using the computational hits from the previous step, specifically molecules II-16 and II-23, as query molecules. This similarity search was performed using the Zinc database,^29^ resulting in a new molecular database composed of 108 molecules: 69 similar to molecule II-16 and 39 similar to molecule II-23. The expanded database was then subjected to a new docking protocol with the four amidases. The results are presented in Table S3 and Figure 1B.

**Scheme 1:**
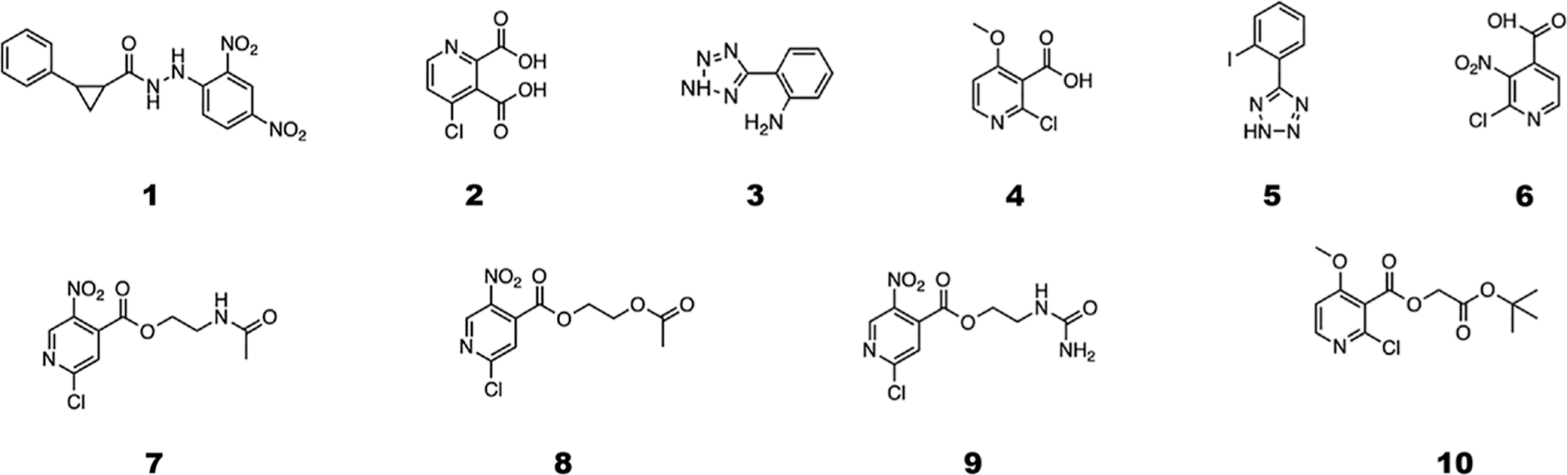
Selected Final Molecules Resulting from In Silico Analysis

The final selection of molecules for experimental assessment was determined based on their overall docking score across the four amidases, as well as their druggable properties, availability, and cost. The selected top ten molecules are: II-27 (**2**), III-30 (**3**), II-16 (**4**), III-33 (**5**), II-23 (**6**), II-23–30 (**7**), II-23–20 (**8**), II-23–26 (**9**) and I-16–49 (**10**) (Scheme 1). The number in parentheses represents the new identifiers that will be used henceforth in the text.

We also focused on identifying chemical cores with structural similarity to the substrate. Initially, we performed a docking validation by redocking the Ami1-dipeptide product (L-Ala-iso-D-Gln) into the catalytic pocket of Ami1. The root-mean-square deviation (RMSD) between the cocrystallized (PDB: 4M6G)^16^ and redocked dipeptide conformers was 1.0 Å, with a binding affinity −5.8 kcal/mol, confirming the validity of the docking protocol (Figure S4).

We then screened and docked the database compounds against the target protein using AutoDock Vina.^30^ The ten compounds with the lowest binding energies were selected as potential candidates. These compounds were further filtered based on their drug-likeness, bioavailability and lead-likeness as shown in Table S4. Compounds with a bioavailability score of less than 0.5, lacking lead-likeness, or violating the Lipinski et al.,^31^ Veber et al.^32^ and Muegge et al.,^33^ rules were excluded. As a result, the compound ZINC05003646 (**1**), with a binding free energy of −7.4 kcal/mol, was selected as the best potential candidate for the further experimental studies (Scheme 1).

**Figure 2:**
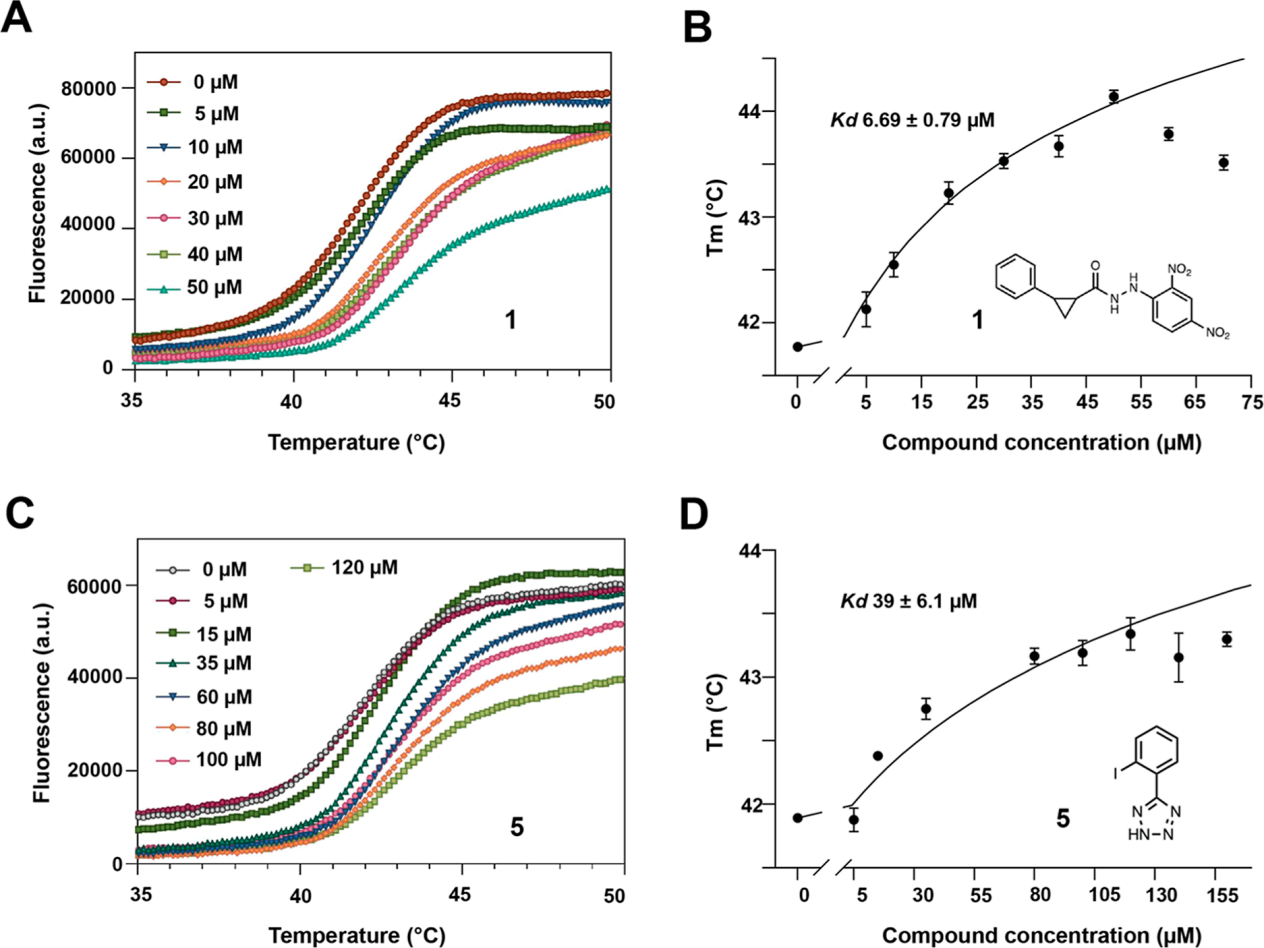
Differential scanning fluorimetry assay. (A,C) Representative thermal stability curves of Ami1 at different concentrations of compounds **1** and **5**, respectively. Each color represents a different compound concentration. (B,D) Dose–response curves for compound **1** and **5**. All experiments were performed in triplicated.

The final set of compounds selected (Scheme 1) was evaluated using differential scanning fluorimetry (DSF). Despite testing various constructs of Ami2–Ami3 and using different expression strains, we consistently obtained very low quantities of soluble proteins, which hindered the interaction experiments. Consequently, experimental interaction was only confirmed with Ami1. Of the ten compounds tested, compounds **1** and **5** altered protein stability, increasing the melting temperature (*T*_m_) of the amidase by approximately 2 °C compared to the *T*_m_ of the protein in the absence of any compound, as shown in the thermal shift graphs (Figure 2). For these two compounds, the curves shifted to the right as the concentration of the compounds increased (Figure 2A,C). The dose–response curves were fitted as detailed in the Methods section. The calculated dissociation constant (*K*_d_) for compound **1** was 6.69 ± 0.79 μM, and for compound **5**, it was 39 ± 6.1 μM. It is important to note that these compounds have completely different structures, which may explain the variation in *K*_d_ values by an order of magnitude (Figure 2B,C).

**Scheme 2:**
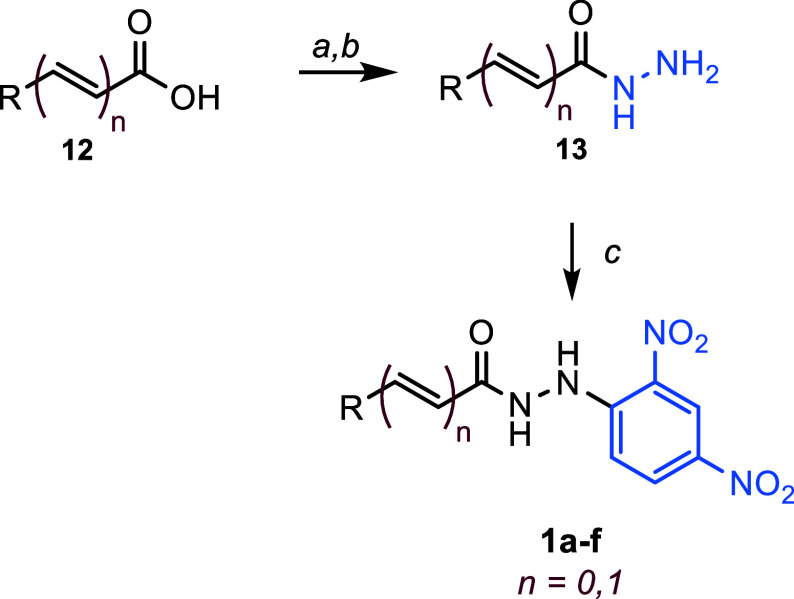
Synthesis of Hydrazides **1a–f** Reagents and conditions: (a) CDI, THF, 45 °C, 2 h, then *t*-butylcarbazate, 45 °C, 14 h; (b) TFA, 2 h, 35 °C; (c) 2,4-dinitrochlorobenzene, EtOH, 35 °C, 2–3 h.

In the light of the findings presented above, we became interested in preparing a small collection of derivatives based on hit compound **1**. As an initial strategy, we attempted the coupling of 2,4-dinitrophenylhydrazine with activated acids but were unable to isolate the corresponding carbohydrazides. Therefore, we modified the synthetic protocol: first, we prepared the carbohydrazide moiety, which then served as the nucleophile in a subsequent S_N_Ar mechanism (Scheme 2). Briefly, different carboxylic acids (**12**) were reacted with 1,1-carbonyldiimidazole (CDI) and then coupled with *t*-butylcarbazate. After Boc cleavage with TFA, the aromatic nucleophilic substitution between hydrazides (**13**) and 2,4-dinitrochlorobenzene afforded the desired products **1a–f**.

**Scheme 3:**
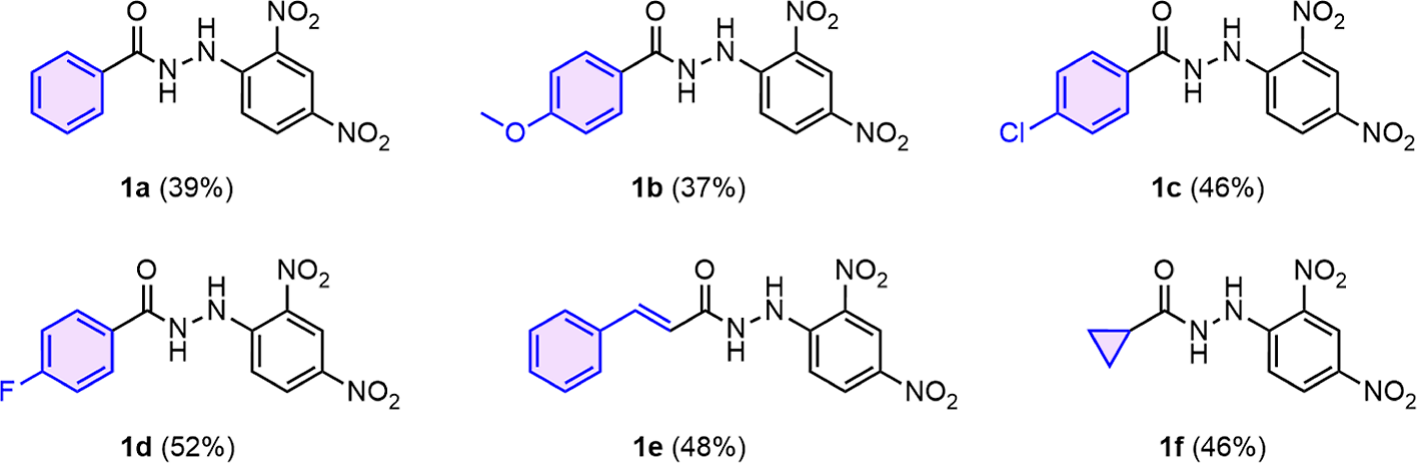
Structure of Hydrazides **1a–f** The yield for the three-step sequence is shown in parentheses.

The collection initially investigated the impact of removing the cyclopropyl bridge observed in the virtual lead **1**. We then decorated the phenyl ring with electron-donating (**1b**) and electron-withdrawing (**1c** and **1d**) groups. Next, we analyzed the effect of replacing the cyclopropyl linker with a double bond (**1e**). Finally, we eliminated the phenyl ring while retaining the cyclopropyl ring (**1f**) (Scheme 3).

**Figure 3:**
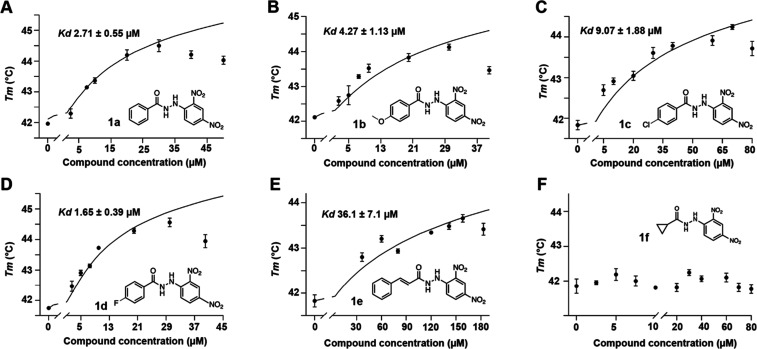
Dose–response curves for derivatives **1a–1f**. All experiments were performed in triplicated.

The different chemical modifications in the carbohydrazide core of each synthetic derivate (**1a–1f**) were evaluated in terms of their affinity to Ami1 by calculating the *K*_d_. As shown in Figure 3, the presence of the cyclopropyl ring is not a prerequisite for protein-compound interaction. Moreover, when the cyclopropyl ring is replaced by a phenyl ring alone (Figure 3A) or by a phenyl ring attached to electron-donating or electron-withdrawing groups (Figure 3B–D), an increase in affinity was observed. In contrast, for derivative **1e**, replacing the cyclopropyl linker with a double-bound caused a 10-fold increase in *K*_d_ compared with chemical derivates **1a–1d** (Figure 3E), thereby lowering affinity to Ami1. Interestingly, synthetic derivative **1f**, in which the cyclopropyl ring was maintained but the phenyl ring was removed, showed no interaction with Ami1 (Figure 3F). This observation suggests that, in addition to the carbohydrazide core, an electron donor such as the phenyl ring may be necessary for the interaction with the amidase.

**Figure 4:**
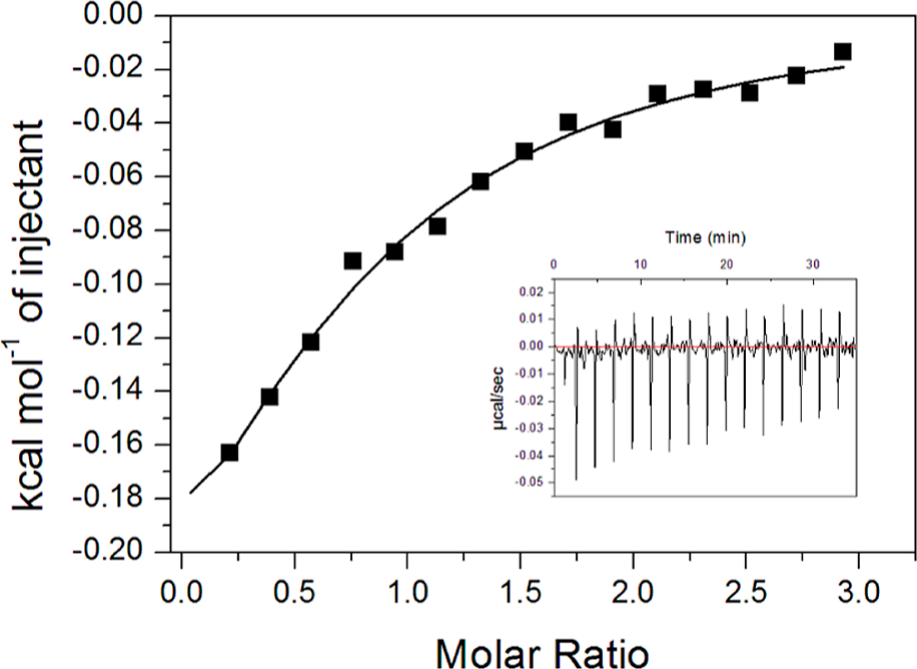
ITC assay for the binding affinity between Ami1 and compound **5**. The plot displays the raw data of heat pulses resulting from the titration of Ami1 in the calorimetric cell with injections of compound **5**. The inset plot shows the integrated heat of the pulses. The data were fitted to a single binding-site model, with the solid black line represent the best fit.

Due to the low solubility of the evaluated compounds, we characterized the binding capacity of only compound **5** to Ami1 using isothermal titration calorimetry (ITC).^34^Figure 4 shows the best nonlinear regression fitting to a single binding-site model. The fitting suggests a single binding site in Ami1 with a stoichiometry of 0.7. As summarized in Table 1, compound **5** exhibits a moderate affinity for Ami1, driven primarily by enthalpic contributions, likely due to the desolvation of both the binding site and the compound.^35,36^ This will be discussed in more detail later.

**Table 1:** Thermodynamics Parameters of Binding Analyzed by ITC

*K*_b_ (M^–1^ × 10^4^)	Δ*H*_b_ (kcal/mol)	Δ*G*_b_ (kcal/mol)	–*T*Δ*S*_b_ (kcal/mol)	*n*
2.60 ± 0.53	–0.37 ± 0.08	–6.02	5.65	0.7 ± 0.1

Despite our efforts, we have not yet been able to obtain crystallographic complexes of Ami1 with compound **1** and its derivates, likely due to the low solubility of these compounds. However, a blind docking procedure successfully placed compound **1** and derivative **1a** within the catalytic cavity of Ami1, as expected, since they were designed as potential mimics of natural amidase ligand. A comparison of these docking results with Ami1-dipeptide product (L-Ala-iso-D-Gln) PDB 4M6G^16^ revealed a structural overlap between these compounds and the dipeptide (Figure S5).

As previously stated, three compounds identified by Küssau and collaborators were proposed as potential inhibitors of Ami1_Mab_ from *M. abscessus*.^23^ This protein shares 64% identity with Ami1_Mt_ from *M. tuberculosis* (Figure S6A). One of these inhibitors features an amine group (inhibitor II) instead of an iodine atom found in compound **5** proposed in the present work (Figure S6B). Additionally, inhibition of the hydrolytic activity of Ami1_Mab_ was observed starting at 800 μM of inhibitor II, and in silico docking studies suggested binding at the active site of Ami1_Mab_.^23^

**Figure 5:**
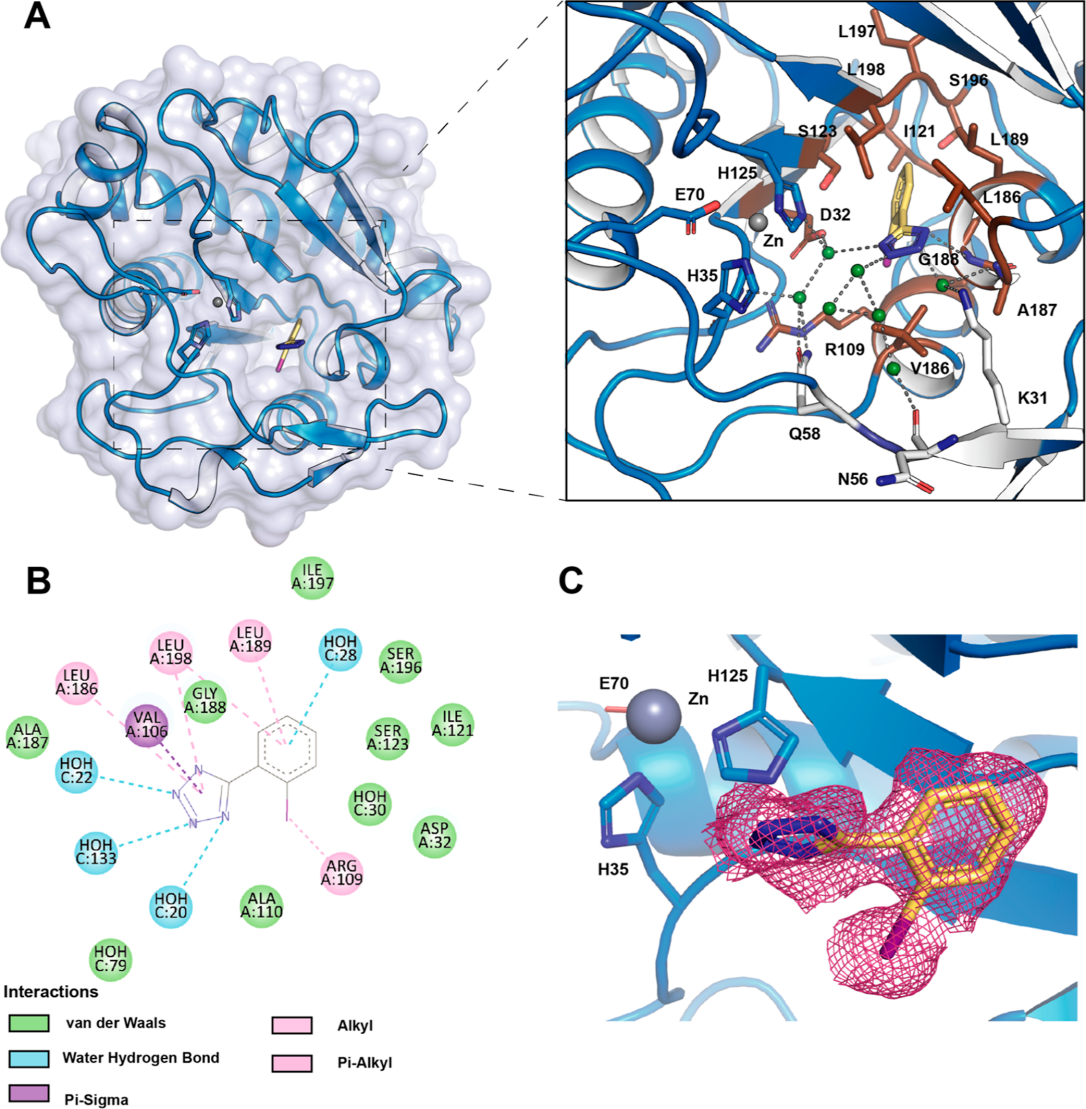
Crystallographic structure of the Ami1–5 complex. (A) The left panel shows a surface representation of Ami1 with compound **5** positioned in the catalytic groove. The right panel provides a close-up view of the residues interacting with compound **5**. In both panels, the catalytic triad amino acids are shown in blue sticks, residues interacting with compound **5** in brown sticks, amino acids with water-mediated interactions in white, compound **5** in yelow, the zinc atom is depicted as a gray sphere, and water molecules are shown as green spheres. (B) Schematic representation of the interactions between Ami1 and compound **5**, generated using Molecular Operating Environment (MOE) software. (C) Polder omit map (Fo-Fc omit map) for compound **5**, contoured at 3.0 σ, shown in raspberry.

In this work, we obtained the 3-D structure of the Ami1-**5** complex at atomic resolution (1.45 Å) (Table S5). Compound **5** was localized within the catalytic groove (Figure 5A), positioned at 4.3 Å from residue His125, which is part of the coordination triad responsible for binding Zn^2+^, along with residues His35 and Glu70 in the active site. The importance of the Zn^2+^ ion in Ami1 activity was demonstrated in a study where treatment of the enzyme with 10 mM EDTA abolished its hydrolytic activity.^16^

Compound **5** is positioned within a hydrophobic core composed of residues Leu186, Leu189, Leu197 and Leu198, Val186, Ala187, Gly188 and Ile121 (Figure 5A). The phenyl ring is stabilized by π–alkyl interactions with Leu189 and Leu198, and van der Waals interactions with Asp32, Ile121, Ser123, and Ser196. The tetrazole ring is stabilized by Val106 (which adopts a double conformation) through a π–σ interaction, π–alkyl interactions with Leu196 and Leu198, and hydrogen bonds formed with the main chain of Gly188 and three water molecules. Finally, the iodine atom engages in an alkyl interaction with residue Arg109 (Figure 5A,B). The extraordinary quality of the electron density map for compound **5** is shown in Figure 5C.

Interestingly, a network of water-mediated interactions stabilizes the tetrazole ring of compound **5**. These water molecules are positioned in the catalytic site, between the hydrophobic core and the coordination zinc triad (Figure 5A). We also resolved the 3-D structure of apo Ami1 at 1.2 Å resolution, where five water molecules occupy the position where compound **5** binds, similar to those observed in previously reported apo Ami1 structure (PDB: 4LQ6)^17^ (Figure S7). This suggests that the binding of compound **5** to the protein results in the simultaneous desolvation of these water molecules, meaning they are released upon compound **5** binding. This displacement could affect the thermodynamic signature of the binding event, potentially making it entropically favorable, which might be offset by a loss of enthalpy^35,36^ (Table 1).

A structural superposition of the Ami1-**5** complex obtained in this work with the previously reported Ami1-dipeptide complex (l-alanine-iso-d-glutamine, PDB: 4M6G)^16^ shows an overlap in the catalytic cavity between compound **5** and the dipeptide. There is only a 0.7 Å distance between a nitrogen atom (N06) of the tetrazole ring in compound **5** and an oxygen atom (0 × 10^1^) of iso-d-glutamine (Figure S8). This suggests that the presence of compound **5** in the cavity may hinder substrate binding, and, consequently, inhibit the hydrolysis reaction.

## Conclusion

Using virtual screening, we identified two initial compounds that demonstrated experimental interaction with the amidase Ami1. Building on one of these compounds, we proposed a small collection of chemical derivatives that showed improved affinity by this amidase. Additionally, we reported the crystallographic structure of the Ami1-**5** complex, which allowed us to observe the molecular interactions of one of these compounds within the catalytic site. These results represent a preliminary approach in proposing a new target and contributing to drug design against tuberculosis.

## Methods

### Chemistry

#### General Procedures

Reagents and solvents were acquired from Merck and Química Rique. NMR spectra were recorded on a Bruker Avance III 400 MHz spectrometer using CDCl_3_ and DMSO-*d*_6_. Mass spectra (MS) and high-resolution mass spectra (HRMS) were measured using a Jeol JMS-T100LC, The AccuTOF mass spectrometer with the DART (Direct Analysis in Real Time), ionization technique.

#### General Method for the Synthesis of Hydrazides **1a–f**

In a round-bottom flask, the corresponding carboxylic acid (0.69 mmol) and carbonyldiimidazole (CDI) (0.77 mmol) were dissolved in THF (3.5 mL) and heated at 45 °C for 2 h. Once the starting material was consumed, *t*-butylcarbazate (0.63 mmol) was added and allowed to react for 14 h at 45 °C. The solvent was removed under vacuum and then redissolved in AcOEt. The organic solution was washed with a 10% aqueous HCl solution. The organic phase was dried over Na_2_SO_4_, evaporated, and treated with trifluoracetic acid (3.15 mmol), stirred for 2 h at 35 °C. After Boc cleavage was complete, the reaction mixture was diluted with AcOEt and washed several times with a 10% aqueous NaHCO_3_ solution. The organic phase was dried over Na_2_SO_4_ evaporated and used in the final step without purification. After dissolving the remnant of the last step with EtOH (2 mL), 2,4-dinitrochlorobenzene (0.6 mmol) was added, and the mixture was heated at 50 °C for 2–4 h. The product was then purified by flash column chromatography.

#### *N*′-(2,4-Dinitrophenyl)benzohydrazide (**1a**)

Obtained as a yellow solid in 39% yield after purification by flash column chromatography (Hex–AcOEt 7:3). ^1^H NMR (CDCl_3_ and DMSO-*d*_6_, 400 MHz) δ: 10.99 (br s, 1H), 10.06 (s, 1H), 8.94 (dd, *J* = 2.8 and 0.8 Hz, 1H), 8.23 (dd, *J* = 9.6 and 2.4 Hz, 1H), 7.93–7.91 (m, 2H), 7.56–7.52 (m, 1H), 7.47–7.43 (m, 2H), 7.28 (d, *J* = 9.6 Hz, 1H); ^13^C NMR (CDCl_3_ and DMSO-*d*_6_, 100 MHz) δ: 165.6, 147.7, 136.2, 131.2, 130.6, 129.1, 128.9, 127.4, 126.6, 122.1, 114.3; MS (DART^+^) *m*/*z*: 303 [M + H]^+^; HRMS *m*/*z* calcd for ^12^C_13_^1^H_11_^14^N_4_^16^O_5_ [M + H]^+^, 303.07294; found, 303.07300.

#### *N*′-(2,4-Dinitrophenyl)-4-methoxybenzohydrazide (**1b**)

Obtained as a dark yellow solid in 37% yield after purification by flash column chromatography (Hex–AcOEt 7:3 and then 1:1). ^1^H NMR (DMSO-*d*_6_, 400 MHz) δ: 10.91 (br s, 1H), 10.19 (s, 1H), 8.87 (d, *J* = 2.8 Hz, 1H), 8.29 (dd, *J* = 9.6 and 2.8 Hz, 1H), 7.93–7.89 (AÁBB́, 2H), 7.25 (d, *J* = 9.6 Hz, 1H), 7.07–7.04 (AÁBB́, 2H), 3.81 (s, 3H); ^13^C NMR (DMSO-*d*_6_, 100 MHz) δ: 166.3, 162.9, 149.2, 137.2, 130.6, 130.2, 130.1, 124.2, 123.6, 115.9, 114.3, 55.9; MS (DART^+^) *m*/*z*: 333 [M + H]^+^; HRMS *m*/*z* calcd for ^12^C_14_^1^H_13_^14^N_4_^16^O_6_ [M + H]^+^, 333.08351; found, 333.08367.

#### 4-Chloro-*N*′-(2,4-dinitrophenyl)benzohydrazide (**1c**)

Obtained as a yellow solid in 46% yield after purification by flash column chromatography (Hex–AcOEt 7:3). ^1^H NMR (DMSO-*d*_6_, 400 MHz) δ: 11.12 (br s, 1H), 10.23 (s, 1H), 8.87 (d, *J* = 2.8 Hz, 1H), 8.29 (dd, *J* = 9.6 and 2.8 Hz, 1H), 7.95–7.92 (AÁBB́, 2H), 7.62–7.58 (AÁBB́, 2H), 7.29 (d, *J* = 9.6 Hz, 1H); ^13^C NMR (DMSO-*d*_6_, 100 MHz) δ: 165.9, 148.9, 137.7, 137.3, 131.0, 130.7, 130.3, 130.0, 129.2, 123.6, 115.9; MS (DART^+^) *m*/*z*: 337 [M + H]^+^; HRMS *m*/*z* calcd for ^12^C_13_^1^H_10_^35^Cl_1_^14^N_4_^16^O_5_ [M + H]^+^, 337.03397; found, 337.03405.

#### *N*′-(2,4-Dinitrophenyl)-4-fluorobenzohydrazide (**1d**)

Obtained as a yellow solid in 52% yield after purification by flash column chromatography (Hex–AcOEt 1:1). ^1^H NMR (DMSO-*d*_6_, 400 MHz) δ: 11.06 (br s, 1H), 10.27 (s, 1H), 8.85 (d, *J* = 2.8 Hz, 1H), 8.27 (dd, *J* = 9.6 and 2.4 Hz, 1H), 8.03–7.98 (m, 2H), 7.40–7.33 (m, 2H), 7.29 (d, *J* = 9.6 Hz, 1H); ^13^C NMR (DMSO-*d*_6_, 100 MHz) δ: 165.0, 164.5 (d, *J* = 248.7 Hz), 148.5, 136.8, 130.4 (d, *J* = 9.2 Hz), 130.2, 129.7, 128.6 (d, *J* = 2.8 Hz), 123.2, 115.7 (d, *J* = 21.9 Hz), 115.6; MS (DART^+^) *m*/*z*: 321 [M + H]^+^; HRMS *m*/*z* calcd for ^12^C_13_^1^H_10_^19^F_1_^14^N_4_^16^O_5_ [M + H]^+^, 321.06352; found, 321.06345.

#### *N*′-(2,4-Dinitrophenyl)cinnamohydrazide (**1e**)

Obtained as an orange solid in 48% yield after purification by flash column chromatography (Hex–AcOEt 7:3). ^1^H NMR (DMSO-*d*_6_, 400 MHz) δ: 10.75 (br s, 1H), 10.18 (s, 1H), 8.85 (d, *J* = 2.8 Hz, 1H), 8.29 (dd, *J* = 9.6 and 2.4 Hz, 1H), 8.64–7.61 (comp, 2H), 7.58 (d, *J* = 16.0 Hz, 1H), 7.45–7.40 (comp, 3H), 7.20 (d, *J* = 9.6 Hz, 1H), 7.74 (d, *J* = 16.0 Hz, 1H); ^13^C NMR (DMSO-*d*_6_, 100 MHz) δ: 165.7, 148.8, 142.2, 137.4, 134.7, 130.9, 130.7, 130.3, 129.7, 128.5, 123.7, 118.9, 115.9; MS (DART^+^) *m*/*z*: 329 [M + H]^+^; HRMS *m*/*z* calcd for ^12^C_15_^1^H_13_^14^N_4_^16^O_5_ [M + H]^+^, 329.08859; found, 329.08865.

#### *N*′-(2,4-Dinitrophenyl)cyclopropanecarbohydrazide (**1f**)

Obtained as beige solid in 46% yield after purification by flash column chromatography (Hex–AcOEt 7:3). ^1^H NMR (DMSO-*d*_6_, 400 MHz) δ: 10.63 (s, 1H), 10.08 (s, 1H), 8.85 (dd, *J* = 2.6 and 1.6 Hz, 1H), 8.35 (ddd, *J* = 9.6, 2.4, and 1.6 Hz, 1H), 7.17 (dd, *J* = 9.6 and 1.6 Hz, 1H), 1.77–1.71 (m, 1H), 0.87–0.83 (m, 2H), 0.82–0.78 (m, 2H); ^13^C NMR (DMSO-*d*_6_, 100 MHz) δ: 172.6, 148.6, 136.6, 130.3, 129.6, 123.2, 115.3, 12.1, 7.0; MS (DART^+^) *m*/*z*: 267 [M + H]^+^; HRMS *m*/*z* calcd for ^12^C_10_^1^H_11_^14^N_4_^16^O_5_ [M + H]^+^, 267.07294; found, 267.07262.

### Cloning, Expression, and Purification of Ami1

The coding fragment corresponding to residues 25–241of Ami1 was amplified by PCR using specific oligonucleotides and genomic DNA from *M. tuberculosis* H37Rv as the template The fragment was inserted into the vector pCS157 between the restriction sites *Bam*HI and *Hin*dIII, resulting in the plasmid pCS157-Ami1. This vector was transformed into *E. coli* BL21 (DE3)pLysS, and the bacteria containing pCS157-Ami1 were grown in Luria–Bertani medium with 100 μg/mL ampicillin and 25 μg/mL chloramphenicol at 37 °C with agitation until an optical density (OD) of 0.6 was reached. Protein expression was induced by adding 0.2 mM of isopropyl β-d-thiogalactopyranoside (IPTG) and incubating for 16 h at 16 °C. Cells were harvested by centrifugation, resuspended in lysis buffer (50 mM Tris-HCl, pH 8.0, 500 mM NaCl, 10% glycerol and 0.25 mM protease inhibitor PMSF), and lysed by sonication. The lysate was centrifugated at 14,000 rpm for 45 min, and the supernatant was loaded onto a HisTrap column previously equilibrated with buffer A (20 mM Tris-HCl, pH 8.0 and 300 mM NaCl). The fusion protein was eluted using a step gradient of imidazole in buffer A, with concentrations of 20, 50, 100, 150, 250 and 500 mM. Fractions containing the recombinant protein were pooled and dialyzed against 20 mM Tris-HCl, pH 8.0 and 100 mM NaCl. The SUMO protein was cleaved with ULP1 protease for 1 h at room temperature. The reaction mixture was loaded onto a HisTrap column, and the flowthrough containing Ami1 was collected. The purity of the sample was assessed by SDS-PAGE. Finally, the purified protein was dialyzed into buffer (20 mM HEPES, pH 7.5, 100 mM NaCl), concentrated to 13 mg/mL using a Vivaspin Turbo centrifugal filter with a 10 kDa cutoff, and stored at −80 °C until use.

### Differential Scanning Fluorimetry

DSF assays were conducted using a QuantStudio3 real-time PCR system from Thermo Fisher Scientific, with a MicroAmp Fast 96-well reaction plate and covered with the MicroAmp Optical Adhesive Film (both from Thermo Fisher Scientific). The final reaction volume in each well was 20 μL, containing 10 μM Ami1 in buffer (20 mM HEPES pH 7.5, 100 mM NaCl, 1% DMSO) and 5× SYPRO Orange, either in the absence or presence of various concentrations of compounds. As a control, identical experiments were performed without protein. The temperature was increased from 25 to 99 °C at a gradual rate of 0.2 °C per 12.5 s. Data analysis and dissociation constants (*K*_d_) were determined using the Thermott web application (https://thermott.com).^37,38^ Melting curves were exported from Thermott and visualized using the GraphPad Prism program. All experiments were performed by triplicate.

### Protein Crystallization and Structure Determination

Ami1 was crystallized using the sitting-drop vapor diffusion method at 10 mg/mL. A 1 μL drop of protein solution was mixed with 2 μL of the precipitant solution containing 1 M ammonium sulfate, 0.1 M NaCl, 100 mM HEPES (pH 7.5) and 9 mM cupric chloride. The complex Ami1-**5** was obtained by soaking crystals in a solution of 1 mM compound 5 dissolved in the crystallization condition and incubated overnight. The crystals were then cryoprotected in a saturated solution of lithium sulfate and cooled to 100 K. Diffraction data sets were collected at the XALOC beamline at the ALBA synchrotron facility (Barcelona, Spain). Diffraction data were processed using XDS^39^ and Aimless^40^ from CCP4 suite. The structure was refined using Refmac and Phenix and modeled using Coot.^41^ The crystal present one molecule in the asymmetric unit. Values for crystallographic data collection and refinement are included in Table S5. The final coordinates of the Ami1-**5** complex were deposited in the Protein Data Bank (PDB) under accession code 9CUN.

### Isothermal Titration Calorimetry

The binding affinity between Ami1 and compound **5** was determined by ITC experiments using a MicroCal iTC200 instrument (GE Healthcare, Northampton, MA, USA). Both compound **5** and Ami1 were dissolved in 20 mM HEPES, 100 mM NaCl, and 0.7% DMSO at pH 7.5. All samples were degassed under vacuum prior to titration. The Ami1 protein solution (50 μM) was loaded into the syringe and titrated against the compound **5** solution (700 μM) in the ITC sample cell. The titration comprised 16 consecutive injections, starting with an initial 0.5 μL injection, followed by 15 injections of 2.5 μL each, with a 120 s interval between injections. The experiment was conducted at a stirring rate of 750 rpm and a stable temperature of 25 °C. The measured heat changes were corrected for the heat of dilution, which was determined by adding the ligand to a buffer solution under identical conditions and using the same injection scheduled as with the protein sample. Calorimetric data were analyzed by nonlinear regression using Origin 7.0 software (OriginLab, Co., Northampton, MA, USA).

The molecular interaction between Ami 1 and **5** is described by the following thermodynamic relationship
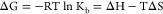
where Δ*G* (kcal/mol) is the standard Gibbs free energy, *R* (kcal/mol K) the gas constant, *T* (K) the temperature, *K*_b_ (M^–1^) the equilibrium binding constant, Δ*H* (kcal/mol) the enthalpy term and −*T*Δ*S* (kcal/mol) the entropic term.

### Molecular Docking

Molecular docking simulations were conducted using the ICM (Internal Coordinate System, Molsoft Inc.) software version 3.9-2b. The tertiary structure of Ami1 was obtained from the Protein Data Bank (PDB) (www.rcsb.org) with the identifier PDB ID: 4LQ6. For Ami2, Ami3, and Ami4, three-dimensional structures were sourced from the AlphaFold Protein Structure Database (AFDB),^26,27^ which employs a deep learning approach to predict high-accuracy protein structures. The models selected for these proteins were chosen based on their confidence scores provided by AlphaFold and accessed through the AFDB platform (https://alphafold.ebi.ac.uk) under the following model IDs: AF-L7N653-F1-v4 for Ami2, AF-Q79F96-F1-v4 for Ami3, and AF-I6Y3Z2-F1-v4 for Ami4. Additionally, these models are accessible via the UniProt database (www.uniprot.org) using their gene sequence IDs: L7N653 for Ami2, Q79F96 for Ami3, and I6Y3Z2 for Ami4. Each 3D structure underwent an energy minimization protocol utilizing the MMFF94s force field. On the other hand, the database of small molecules was prepared by applying protonation at pH 7.0, tautomer assignment, and geometric minimization. Prior to the docking procedure, an interaction site search protocol was conducted using the ICM Pocket Finder tool, integrated within the same software. This tool identifies protein–ligand binding sites by mapping the van der Waals interaction potential of the receptor, thereby highlighting surfaces likely to function as binding sites. The docking scores were obtained directly from the ICM software using a GBSA/MM-type scoring function augmented with a directional hydrogen bonding term. Final docking simulations of selected compounds were performed using AutoDock Vina (version 1.1.2)^30,42^ with default parameters, except for the exhaustiveness value, which was adjusted to 32. During the docking process, all amino acid side chains were treated as rigid. Grid parameters were set with center coordinates *X* = −6.126, *Y* = −8.678, and *Z* = −17.791, and grid dimensions (Å) of *X* = 45, *Y* = 55, and *Z* = 45.

The docking molecular the X-ray structure of apo Ami1 obtained in this study with the selected compounds **1** and **1a** were performed using AutoDock Vina (version 1.1.2)^30,42^ with default parameters, without specifying a binding region. All amino acid side chains were treated as rigid during the docking process. The grid parameters were set as follows: center coordinates *X* = – 6.126, *y* = – 8.678, and *z* = – 17.791, with grid dimensions (Å) of *x* = 45, *y* = 55 and *z* = 45. An exhaustiveness value of 32 was used.

### Drug Likeness Studies

The SwissADME server^43^ was used to calculate the pharmacokinetic and physicochemical properties of the compounds. A range of tests, including the Lipinski et al.,^31^ Veber et al.,^32^ and Muegge et al.,^33^ filters were performed for these ligands.

## Supporting Information Available

Molecular database generated from inhibitors previously described for Ami1_Mab_ from *M. abscesus* (Figure S1 and Table S1); predicted binding sites for the four amidases from *M. tuberculosis* (Figure S2); catalytic residues involved in the activity of the amidases (Ami1–Ami4) from *M. tuberculosis* (Figure S3); results from docking simulations (Tables S2 and S3); structural superposition of the redocked conformer of the L-Ala-iso-D-Gln and the blind dockings of compounds **1** and **1a** with the crystallographic structure of Ami-dipeptide (Figures S4 and S5); the best chemical cores with structural similarity to the substrate of Ami1 (Table S4); comparation of studies between Ami1 from *M. tuberculosis* and *M. abscessus* (Figure S6); crystallographic data (Table S5); the crystal structure of the Ami1-**5** complex and its structural superposition with the dipeptide L-Ala-iso-D-Gln (Figure S7 and S8). ^1^H NMR, ^13^C NMR and HRMS spectra for all synthetic derivates **1a–1f** (PDF)
