# Supplementary material for: Identification of Potential Inhibitors of Mycobacterium tuberculosis Amidases: An Integrated In Silico and Experimental Study
Source: ACS Omega. 2024 Nov 6;9(46):46461–71. doi: 10.1021/acsomega.4c07964 (PMC11579945; doi:10.1021/acsomega.4c07964)
Supplement: Supplementary file 1 — ao4c07964_si_001.pdf [file ao4c07964_si_001.pdf]

## Supporting information

**Identification of Potential Inhibitors of *Mycobacterium tuberculosis* Amidases: An Integrated *In silico* and Experimental Study**

Maciel Rosas-Cruz[1†], Abraham Madariaga Mazón[2†], Carlos D. García-Mejía[1], Eduardo Hernández-Vázquez[1], Homero Gómez-Velasco[1], Eva Jiménez-Faraco[3], Roberto Sealtiel Farías-Gaytán[1], Juan A. Hermoso[3] and Siseth Martínez-Caballero[1*]

[1]Instituto de Química, Universidad Nacional Autonóma de México, A. Universidad 3000, Ciudad Universitaria, C. P. 04510, Ciudad de México, México.

[2]Unidad Mérida del Instituto de Química, Universidad Nacional Autónoma de México, Km. 5.5 Carr. Sierra Papacal - Chuburna Pto. Sierra Papacal, C.P. 97302, Yucatán, México

[3]Department of Crystallography and structural Biology, Instituto de Química-Física "Blas Cabrera", Consejo Superior de Investigaciones Científicas, E-28006, Madrid, Spain.

† These authors contributed equally to this work.

*Corresponding author: siseth.martinez@iquimica.unam.mx

## Index of Figures and Tables

**Figure S1**. References for the generation of a molecular database for identifying amidase inhibitors from *M. tuberculosis*……………………………………………………………………………………………….4

**Figure S2**. Predicted binding sites for the four amidases……………………………………………………….4

**Figure S3**. Superposition of the catalytic residues involved in the activity of the amidases (Ami1-Ami4) from *M. tuberculosis* compared with other amidases.

**Figure S4.** The superimposed structure of the re-docked conformer (pink) of the L-Ala-iso-D-Gln dipeptide over the co-crystallized structure (green) in the active site of Ami1. RMSD value of 1.0 Å……………………………..5

**Figure S5.** Comparation of the structural superposition of the blind dockings with the crystallographic structure Ami1-dipeptide complex………………………………………………………………………………………….5

**Figure S6.** Comparation of studies between Ami1 from *M. tuberculosis* and *M. abscessus*………………………6

**Figure S7.** Crystal structure of the Ami1-**5** complex……………………………………………………………..6

**Figure S8.** Structural superposition of the Ami1-**5** complex with the dipeptide L-alanine-iso-D-Glutamine……...7

**Table S1**. Molecular Database generated from the structural similarity analysis…………………………………...8

**Table S2.** Docking scores for each molecule in the database and for each of the Ami structures………………....12

**Table S3.** Results from docking simulations for the expanded molecular database. ………………………………13

**Table S4.** The structure, free energy of binding, pharmacokinetic and physicochemical properties of the ten best Ami1 inhibitor candidates……………………………………………………………………………………..17

**Table S5.** Crystallographic data…………………………………………………………………………………20

**Spectrum 1**. $^{1}$H-NMR of N'-(2,4-dinitrophenyl)benzohydrazide (**1a**)……………………………………….…21

**Spectrum 2**. $^{13}$C-NMR of N'-(2,4-dinitrophenyl)benzohydrazide (**1a**)…………………………………………22

**Spectrum 3**. HRMS of *N'*-(2,4-dinitrophenyl)benzohydrazide **(1a)**……………………………………………....22

**Spectrum 4**. $^{1}$H-NMR of *N'*-(2,4-dinitrophenyl)-4-methoxybenzohydrazide (**1b**)………………………………..23

**Spectrum 5**. $^{13}$C-NMR of *N'*-(2,4-dinitrophenyl)-4-methoxybenzohydrazide (**1b**)………………………………24

**Spectrum 6**. HRMS of *N'*-(2,4-dinitrophenyl)-4-methoxybenzohydrazide (**1b**)…………………………………24

**Spectrum 7**. $^{1}$H-NMR of 4-chloro-*N'*-(2,4-dinitrophenyl)benzohydrazide (**1c**)…………………………………..25

**Spectrum 8**. $^{13}$C-NMR of 4-chloro-*N'*-(2,4-dinitrophenyl)benzohydrazide (**1c**)…………………………………26

**Spectrum 9**. HRMS of 4-chloro-*N'*-(2,4-dinitrophenyl)benzohydrazide (**1c**)……………………………………26

**Spectrum 10**. $^{1}$H-NMR of *N'*-(2,4-dinitrophenyl)-4-fluorobenzohydrazide (**1d**)…………………………………27

**Spectrum 11**. $^{13}$C-NMR of *N'*-(2,4-dinitrophenyl)-4-fluorobenzohydrazide (**1d**)………………………………...28

**Spectrum 12**. HRMS of *N'*-(2,4-dinitrophenyl)-4-fluorobenzohydrazide (**1d**)……………………………………28

**Spectrum 13**. $^{1}$H-NMR of *N′*-(2,4-dinitrophenyl)cinnamohydrazide (**1e**)…………………………………………...29
**Spectrum 14**. $^{13}$C-NMR of *N′*-(2,4-dinitrophenyl)cinnamohydrazide (**1e**)…………………………………………..30
**Spectrum 15**. HRMS of *N′*-(2,4-dinitrophenyl)cinnamohydrazide (**1e**)……………………………………………..30
**Spectrum 16**. $^{1}$H-NMR of *N'*-(2,4-dinitrophenyl)cyclopropanecarbohydrazide **(1f)**……………………………...31
**Spectrum 17**. $^{13}$C-NMR of *N'*-(2,4-dinitrophenyl)cyclopropanecarbohydrazide **(1f)**……………………………..32
**Spectrum 18**. HRMS of *N'*-(2,4-dinitrophenyl)cyclopropanecarbohydrazide **(1f)**………………………………..32

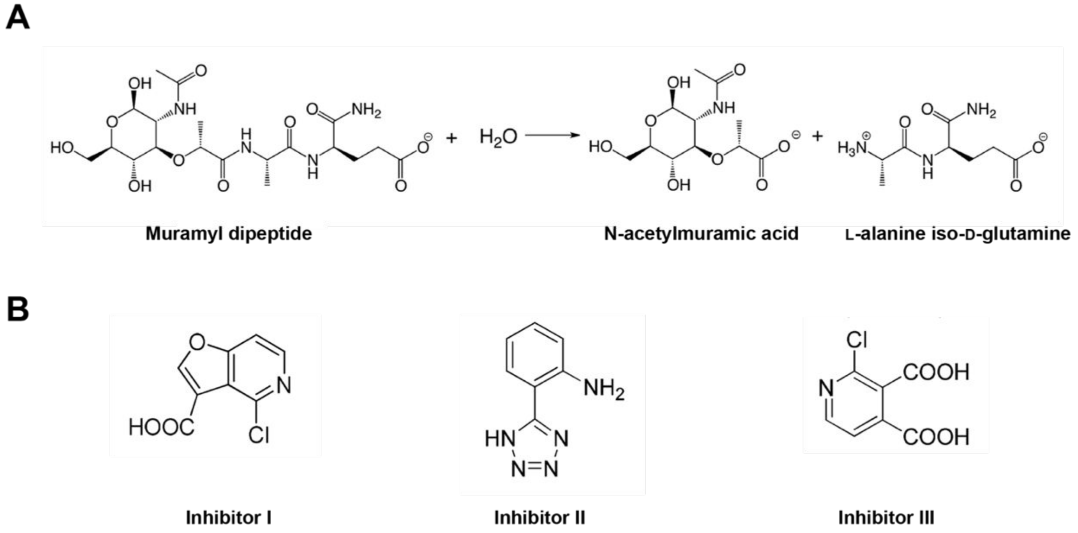


**Figure S1. References for the generation of a molecular database for identifying amidase inhibitors from *M. tuberculosis*.** (A) Schematic representation of the substrate and reaction product of the *N*-acetylmuramyl-L-alanine hydrolases. (B) Chemical structure of inhibitors of Ami1$_{Mab}$ from *M. abscessus* proposed by Küssau and collaborators[1].

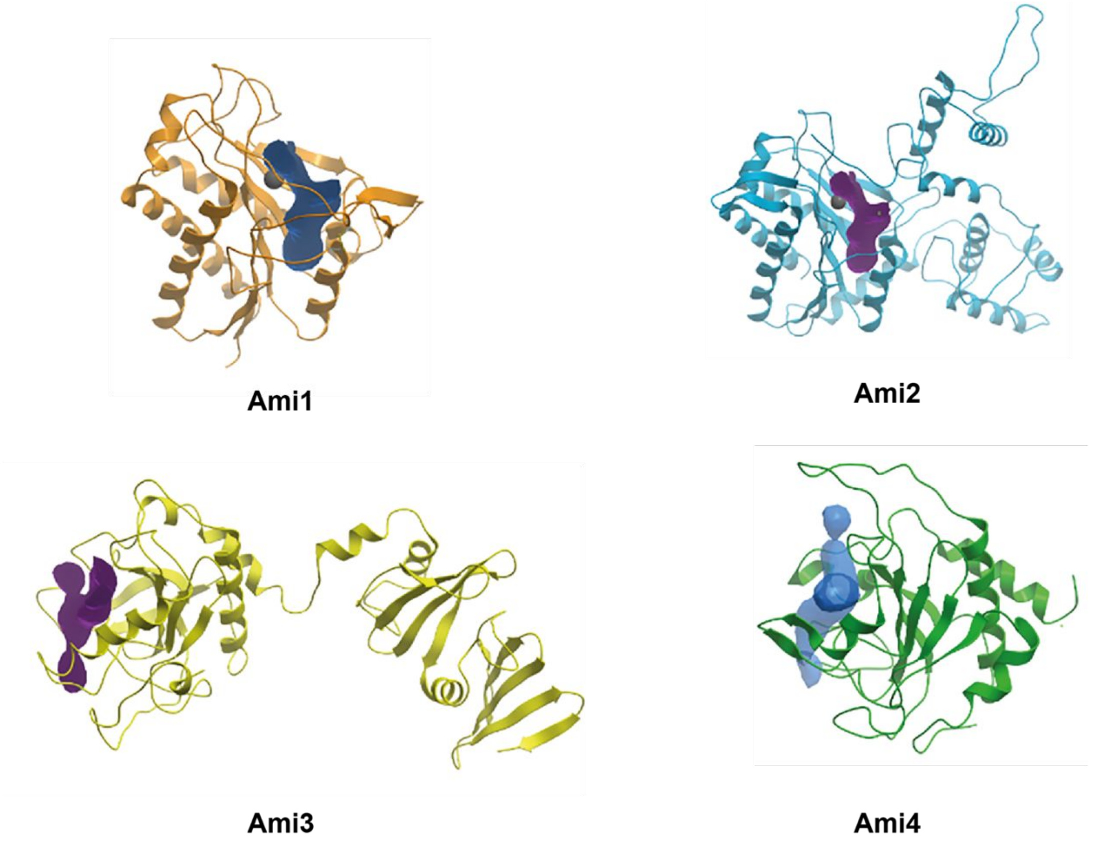


**Figure S2.** Predicted binding sites for the four amidases. The surface representation highlights the regions where the molecular docking study was conducted.

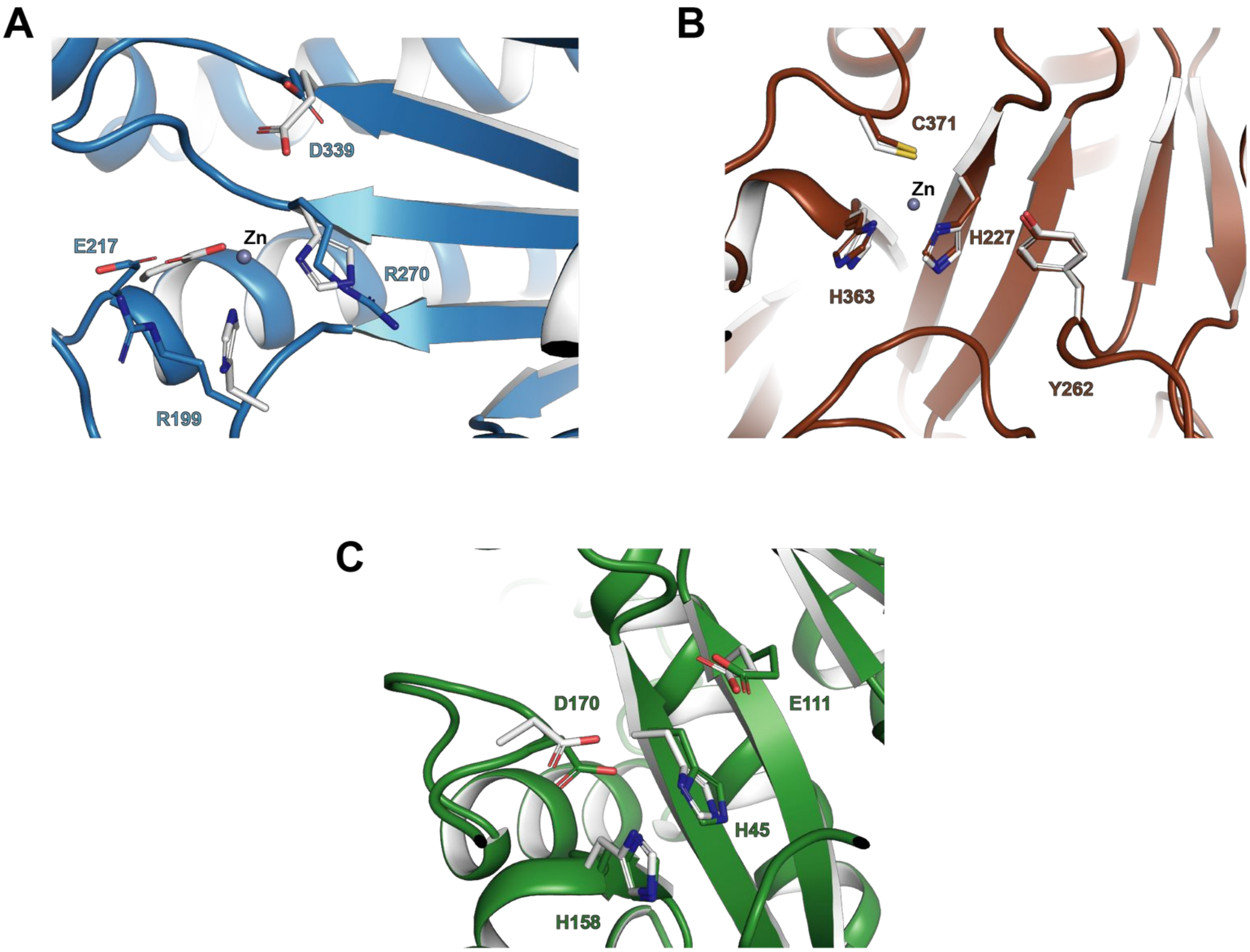


**Figure S3.** Superposition of the catalytic residues involved in the activity of the amidases (Ami1-Ami4) from *M. tuberculosis* compared with other amidases. (A) The AlphaFold model of Ami2 (AF-L7N653) is shown in blue, and the catalytic residues of Ami1 highlighted as white sticks[1]. (B) The AlphaFold model of Ami3 (AF-Q79F96) is shown in brown, and the catalytic residues of the amidase domain of BbtPGRP3 (PDB 4Z8I)[2] from *Branchiostoma belcheri tsingtauense* represented as white sticks. (C) The AlphaFold model of Ami4 (AF-I6Y3Z2) is shown in green, and the catalytic residues of the amidase from *Citrobacter freundii* (PDB 2Y2C)[3] depicted as white sticks.

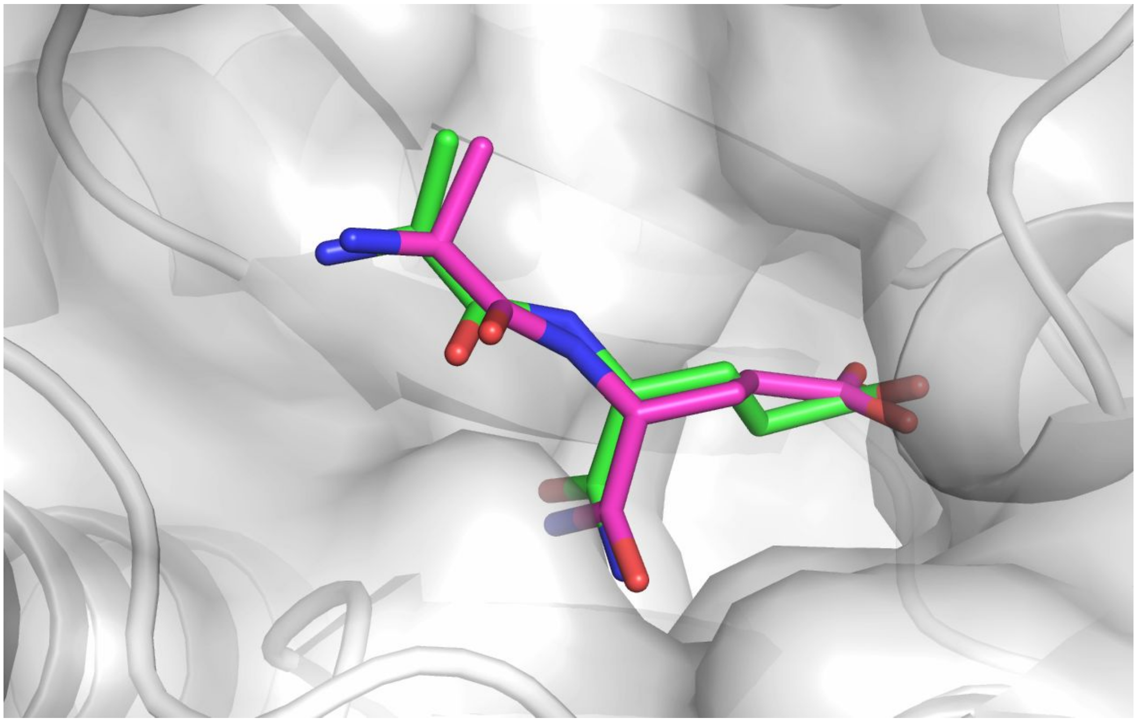

**Figure S4**. The superimposed structure of the re-docked conformer (pink) of the L-Ala-iso-D-Gln dipeptide over the co-crystallized structure (green) in the active site of Ami1 (PDB 4M6G)[4]. RMSD value of 1.0 Å.

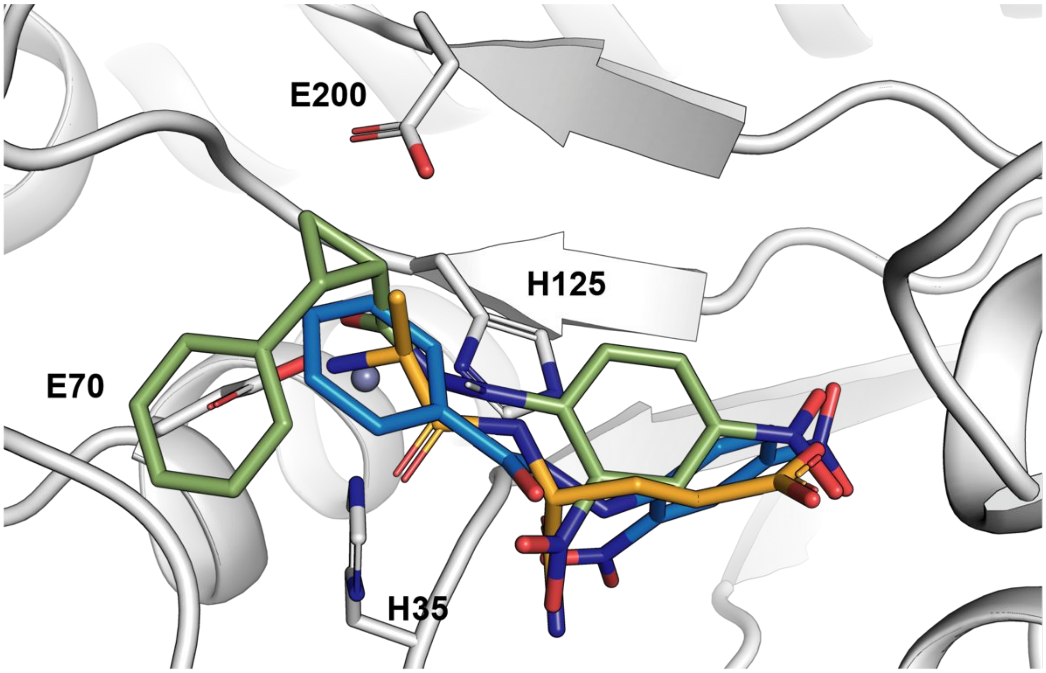


**Figure S5.** Comparation of the structural superposition of the blind dockings with the crystallographic structure Ami1-dipeptide complex (PDB 4M6G)[4]. Amino acids involved in zinc coordination and the catalytic residue E200 are represented in sticks. Compound **1** is shown in green sticks, derivative **1a** in blue sticks, the L-Ala-iso-D-Gln dipeptide in orange, and zinc is depicted as a grey sphere.

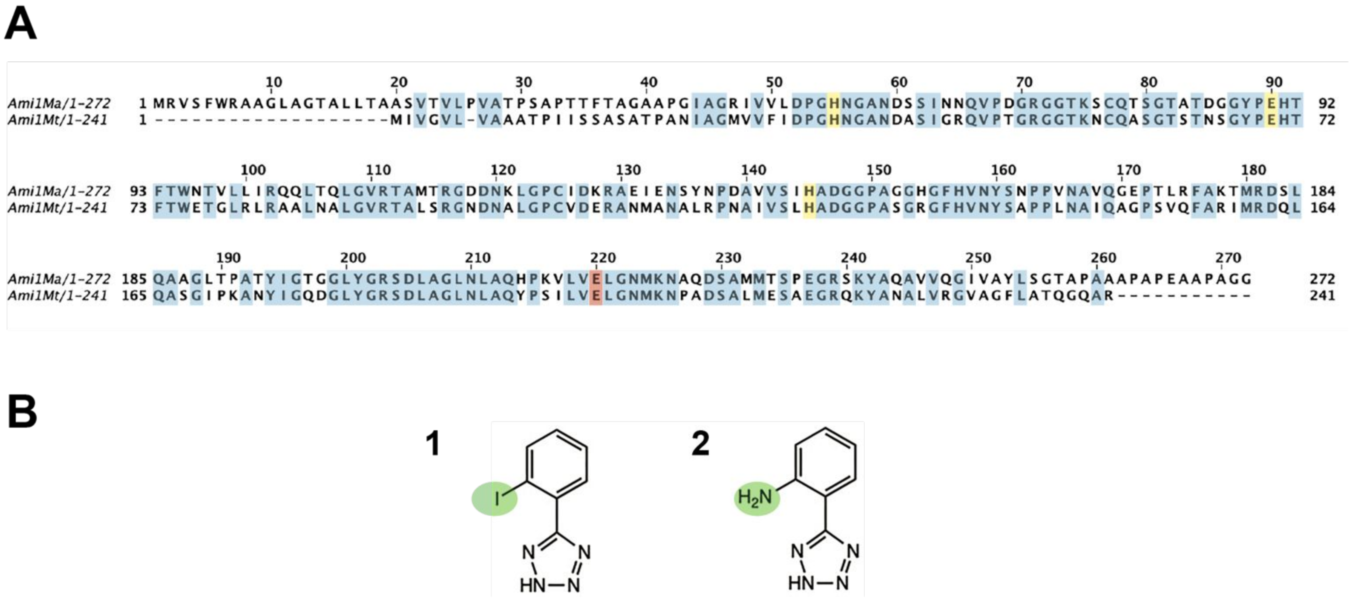


**Figure S6.** Comparation of studies between Ami1 from *M. tuberculosis* and *M. abscessus*. (**A**) Sequence alignment of Ami1 from *M. abscessus* (Ami1$_{\text{Mab}}$) and *M. tuberculosis* (Ami1$_{\text{Mt}}$). Identical residues are shown in blue, residues involved in zinc coordination are shown in yellow, and the catalytic amino acid is shown in orange. (**B**) Tetrazole compounds proposed for the inhibition of Ami1 from *M. tuberculosis* (1) of this study and *M. abscessus* (2) in a previously reported study by Küssau and collaborators[1].

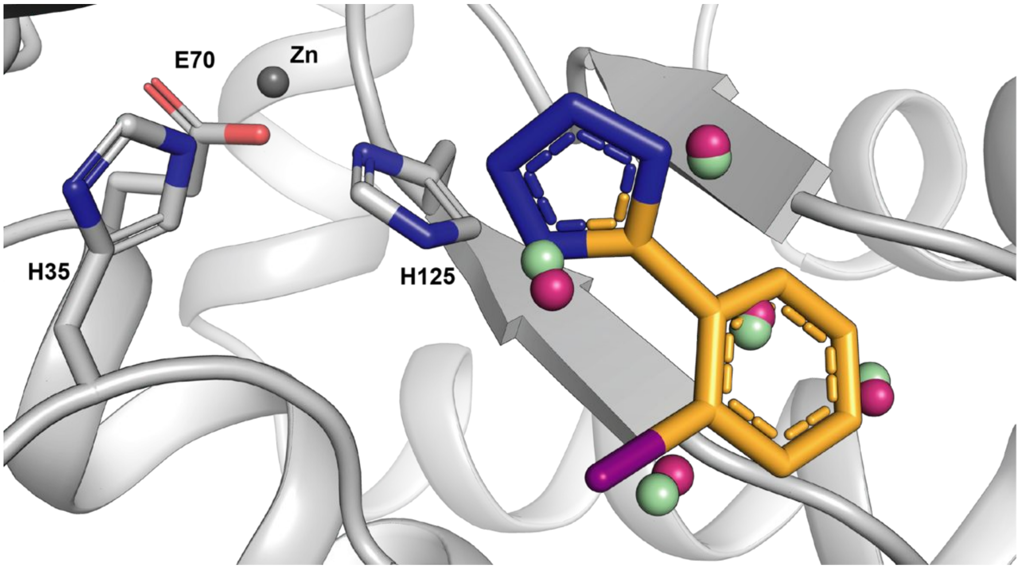


**Figure S7.** Crystal structure of the Ami1-**5** complex. Amino acids participating in zinc coordination are shown as sticks, compound **5** is depicted in orange, waters molecules from apo Ami1 resolved by us are represented as green spheres, and water molecules reported in the apo Ami1 PDB: 4LQ6[5] are shown as pink spheres.

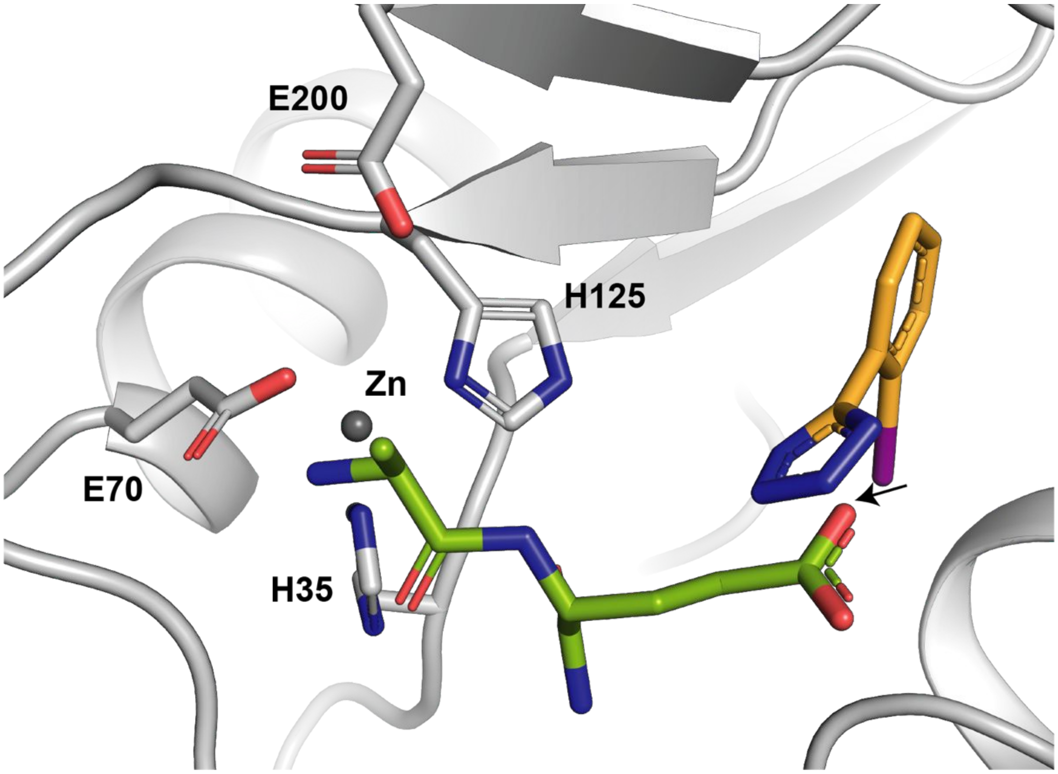


**Figure S8.** Structural superposition of the Ami1-**5** complex with the dipeptide L-alanine-iso-D-Glutamine**.** Residues belonging to the catalytic triad and the catalytic residue E200 are shown as sticks. Compound **5** is depicted in orange, and the reaction product dipeptide from the crystallographic structure (PDB 4M6G)[4] is shown in green. The arrow indicates the distance between the N06 atom of tetrazole and 0E1atom of iso-D-Glutamine.

**Table S1**. Molecular Database generated from the structural similarity analysis. The identifier code in the public Zinc database (Zinc ID), the degree of similarity with the reference inhibitors, and the assigned ID are presented.

| Chemical structure | Zinc ID | Similarity to reference | Database ID |
|---|---|---|---|
| 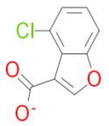 | ZINC000238698447 | Inhibitor I | **I-1** |
| 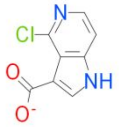 | ZINC000044713147 | Inhibitor I | **I-2** |
| 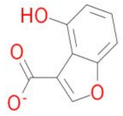 | ZINC000011804035 | Inhibitor I | **I-3** |
| 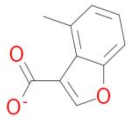 | ZINC000238459969 | Inhibitor I | **I-4** |
| 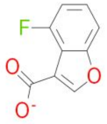 | ZINC000075882418 | Inhibitor I | **I-5** |
| 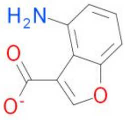 | ZINC000212687734 | Inhibitor I | **I-6** |
| 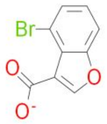 | ZINC000238698429 | Inhibitor I | **I-7** |
| 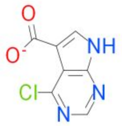 | ZINC000035269901 | Inhibitor I | **I-8** |

| | | | |
|---|---|---|---|
| 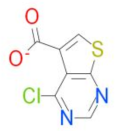 | ZINC000575442078 | Inhibitor I | **I-9** |
| 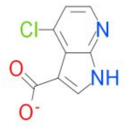 | ZINC000019046167 | Inhibitor I | **I-10** |
| 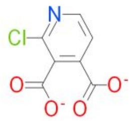 | ZINC000002244263 | Inhibitor II | **II-11** |
| 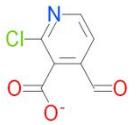 | ZINC000238701697 | Inhibitor II | **II-12** |
| 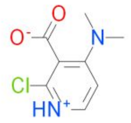 | ZINC000238317920 | Inhibitor II | **II-13** |
| 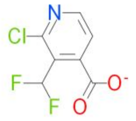 | ZINC000238685572 | Inhibitor II | **II-14** |
| 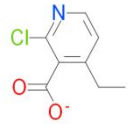 | ZINC000113544211 | Inhibitor II | **II-15** |
| 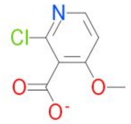 | ZINC000098086085 | Inhibitor II | **II-16** |
| 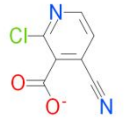 | ZINC000260620677 | Inhibitor II | **II-17** |

| | | | |
|---|---|---|---|
| 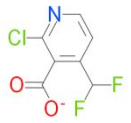 | ZINC000307463019 | Inhibitor II | **II-18** |
| 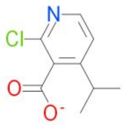 | ZINC000219831856 | Inhibitor II | **II-19** |
| 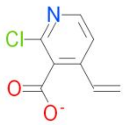 | ZINC000710037554 | Inhibitor II | **II-20** |
| 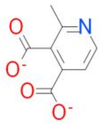 | ZINC000140611326 | Inhibitor II | **II-21** |
| 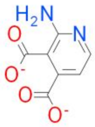 | ZINC000095769036 | Inhibitor II | **II-22** |
| 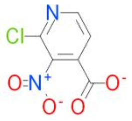 | ZINC000004473036 | Inhibitor II | **II-23** |
| 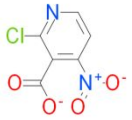 | ZINC000217775262 | Inhibitor II | **II-24** |
| 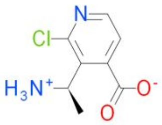 | ZINC000238413726 | Inhibitor II | **II-25** |
| 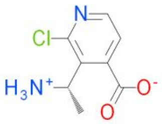 | ZINC000238804732 | Inhibitor II | **II-26** |

| | | | |
|---|---|---|---|
| 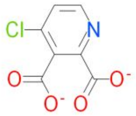 | ZINC000047818720 | Inhibitor II | **II-27** |
| 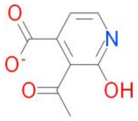 | ZINC000095773813 | Inhibitor II | **II-28** |
| 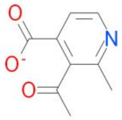 | ZINC000306720577 | Inhibitor II | **II-29** |
| 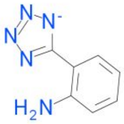 | ZINC000004344222 | Inhibitor III | **III-30** |
| 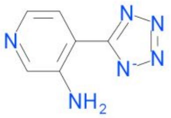 | ZINC000103635302 | Inhibitor III | **III-31** |
| 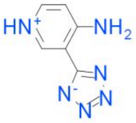 | ZINC000095251990 | Inhibitor III | **III-32** |
| 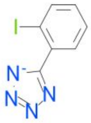 | ZINC000022116517 | Inhibitor III | **III-33** |
| 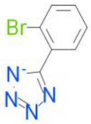 | ZINC000000085629 | Inhibitor III | **III-34** |
| 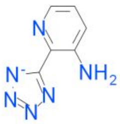 | ZINC000095220031 | Inhibitor III | **III-35** |

**Table S2**. Docking scores for each molecule in the database and for each of the Ami structures.

| ID | Docking scores | | | |
|---|---|---|---|---|
| | Ami1 | Ami2 | Ami3 | Ami4 |
| **I-1** | -19.79 | -11.72 | -15.97 | -17.13 |
| **I-2** | -18.52 | -11.43 | -17.13 | -14.21 |
| **I-3** | -17.45 | -12.16 | -14.89 | -14.56 |
| **I-4** | -18.32 | -11.14 | -14.06 | -13.20 |
| **I-5** | -16.03 | -11.08 | -15.93 | -16.90 |
| **I-6** | -17.00 | -9.99 | -13.93 | -15.49 |
| **I-7** | -19.95 | -11.79 | -15.97 | -17.63 |
| **I-8** | -15.39 | -11.01 | -15.52 | -14.36 |
| **I-9** | -19.81 | -11.35 | -19.86 | -10.77 |
| **I-10** | -16.63 | -11.07 | -16.23 | -14.31 |
| **II-11** | -16.17 | -13.42 | -16.94 | -14.25 |
| **II-12** | -24.20 | -13.19 | -16.29 | -14.11 |
| **II-13** | -15.67 | -11.42 | -15.17 | -12.47 |
| **II-14** | -27.49 | -9.20 | -14.64 | -14.91 |
| **II-15** | -18.96 | -12.39 | -18.08 | -15.35 |
| **II-16** | -24.57 | -15.44 | -15.82 | -15.14 |
| **II-17** | -4.77 | -15.65 | -22.62 | -16.41 |
| **II-18** | -21.00 | -13.19 | -15.72 | -13.29 |
| **II-19** | -21.91 | -15.43 | -16.20 | -13.85 |
| **II-20** | -25.97 | -15.25 | -18.03 | -12.93 |
| **II-21** | -15.21 | -12.78 | -15.65 | -13.46 |
| **II-22** | -22.03 | -9.70 | -12.56 | -11.72 |
| **II-23** | -22.83 | -13.85 | -14.49 | -14.37 |
| **II-24** | -4.49 | -10.12 | -13.78 | -12.01 |
| **II-25** | -13.41 | -15.81 | -15.46 | -10.86 |
| **II-26** | -12.05 | -8.81 | -12.76 | -9.50 |
| **II-27** | -13.92 | -15.32 | -15.72 | -15.78 |
| **II-28** | -17.30 | -15.18 | -17.05 | -13.76 |
| **II-29** | -16.79 | -13.50 | -13.52 | -13.77 |
| **III-30** | -12.75 | -8.65 | -18.56 | -13.18 |

| | | | | |
|---|---|---|---|---|
| **III-31** | -15.54 | -8.34 | -19.05 | -11.53 |
| **III-32** | -11.55 | -10.71 | -16.11 | -10.20 |
| **III-33** | -16.00 | -11.23 | -18.50 | -11.46 |
| **III-34** | -15.31 | -10.35 | -17.72 | -11.16 |
| **III-35** | -11.17 | -7.44 | -18.31 | -11.73 |

**Table S3**. Results from docking simulations for the expanded molecular database. Docking scores are provided for Ami1, Ami2, Ami3 and Ami4.

| ID | Docking score | | | |
|---|---|---|---|---|
| | Ami1 | Ami2 | Ami3 | Ami4 |
| **I-16-1** | -11.906144 | -15.072785 | -19.985497 | -12.93699 |
| **I-16-2** | -8.431624 | -12.763123 | -17.240889 | -15.357023 |
| **I-16-3** | -11.624475 | -10.807748 | -19.018122 | -10.308325 |
| **I-16-4** | -8.303764 | -13.6712 | -19.639727 | -16.743786 |
| **I-16-5** | -13.941184 | -13.976061 | -18.626034 | -9.608711 |
| **I-16-6** | -6.454287 | -13.562113 | -18.580994 | -12.0611 |
| **I-16-7** | -8.197291 | -14.340539 | -15.151642 | -16.120478 |
| **I-16-8** | -8.253846 | -13.724311 | -14.794104 | -13.12509 |
| **I-16-9** | -8.180942 | -13.577089 | -19.998537 | -9.437921 |
| **I-16-10** | -7.482257 | -14.133586 | -17.063694 | -12.433594 |
| **I-16-11** | -9.600781 | -11.825942 | -18.970242 | -8.308308 |
| **I-16-12** | -20.952208 | -14.878175 | -14.373936 | -11.598805 |
| **I-16-13** | -11.97456 | -13.141009 | -14.020084 | -12.989973 |
| **I-16-14** | -15.97729 | -16.3363 | -17.721479 | -15.641242 |
| **I-16-15** | -16.269972 | -11.48264 | -14.636926 | -14.428194 |
| **I-16-16** | -18.073771 | -12.862572 | -14.601289 | -13.543239 |
| **I-16-17** | -8.578367 | -9.760426 | -15.380706 | -11.890309 |
| **I-16-18** | -19.432926 | -14.148577 | -18.75079 | -10.281424 |
| **I-16-19** | -11.950735 | -7.867654 | -19.383636 | -9.852996 |
| **I-16-20** | -9.307451 | -14.688927 | -13.16209 | -14.590058 |
| **I-16-21** | -19.212967 | -16.486818 | -18.88496 | -18.899366 |

| | | | | |
|---|---|---|---|---|
| **I-16-22** | -9.976566 | -12.384093 | -19.145094 | -12.295325 |
| **I-16-23** | -18.185097 | -17.771852 | -11.546852 | -15.347667 |
| **I-16-24** | -8.889184 | -10.959888 | -14.278272 | -16.666782 |
| **I-16-25** | -15.928549 | -12.939595 | -15.493169 | -18.220432 |
| **I-16-26** | -20.450619 | -15.831038 | -11.932866 | -15.37897 |
| **I-16-27** | -16.210987 | -16.120657 | -14.45743 | -15.424141 |
| **I-16-28** | -10.066348 | -10.959629 | -11.510271 | -10.30032 |
| **I-16-29** | -11.145555 | -12.697479 | -19.280491 | -11.483277 |
| **I-16-30** | -6.936958 | -10.931303 | -19.326738 | -8.180623 |
| **I-16-31** | -8.531835 | -19.042446 | -16.263834 | -15.560196 |
| **I-16-32** | -7.768478 | -10.987118 | -14.25406 | -10.526197 |
| **I-16-33** | -10.259928 | -16.916527 | -14.63328 | -14.439139 |
| **I-16-34** | -8.58821 | -13.450591 | -16.624332 | -11.708724 |
| **I-16-35** | -14.605601 | -14.330536 | -10.122657 | -19.017818 |
| **I-16-36** | -10.259188 | -18.382339 | -15.650737 | -7.765926 |
| **I-16-37** | -7.324047 | -10.848977 | -19.268011 | -8.278997 |
| **I-16-38** | -10.2614 | -9.586899 | -15.557203 | -13.745295 |
| **I-16-39** | -20.661646 | -18.823454 | -12.469253 | -16.349323 |
| **I-16-40** | -9.436504 | -16.95219 | -17.559519 | -15.099677 |
| **I-16-41** | -9.693776 | -10.636438 | -15.995455 | -10.181007 |
| **I-16-42** | -17.967718 | -16.442362 | -16.692783 | -18.661604 |
| **I-16-43** | -16.4814 | -12.20607 | -15.966336 | -15.532529 |
| **I-16-44** | -8.572825 | -14.46003 | -11.910646 | -16.960512 |
| **I-16-45** | -12.110548 | -14.420767 | -12.944195 | -13.943836 |
| **I-16-46** | -8.079667 | -10.923815 | -17.416178 | -12.966901 |
| **I-16-47** | -11.795074 | -11.830845 | -16.020296 | -11.90635 |
| **I-16-48** | -11.792165 | -13.769092 | -17.82752 | -10.221072 |
| **I-16-49** | -16.892303 | -17.997469 | -5.798767 | -17.12606 |
| **I-16-50** | -7.433511 | -9.351765 | -17.724932 | -15.313655 |
| **I-16-51** | -7.633653 | -17.033094 | -16.949806 | -16.638161 |
| **I-16-52** | -15.070525 | -17.596592 | -13.114434 | -15.381414 |
| **I-16-53** | -6.185658 | -13.805948 | -15.943485 | -11.063004 |
| **I-16-54** | -7.944918 | -13.623217 | -13.652846 | -11.024628 |

| | | | | |
|---|---|---|---|---|
| **I-16-55** | -19.866776 | -13.366812 | -18.185209 | -19.894758 |
| **I-16-56** | -9.294061 | -11.250409 | -16.105124 | -12.047705 |
| **I-16-57** | -13.258247 | -12.105426 | -15.907857 | -15.709862 |
| **I-16-58** | -13.907969 | -10.980436 | -19.805626 | -12.985453 |
| **I-16-59** | -16.929537 | -16.498684 | -12.705538 | -13.192669 |
| **I-16-60** | -11.148711 | -14.075446 | -16.493904 | -10.870442 |
| **I-16-61** | -10.422639 | -8.527206 | -16.722229 | -10.661522 |
| **I-16-62** | -10.690394 | -10.030159 | -13.881139 | -15.202723 |
| **I-16-63** | -15.387198 | -16.368282 | -9.59064 | -15.215462 |
| **I-16-64** | -16.573614 | -19.047567 | -13.88281 | -13.872242 |
| **I-16-65** | -8.210119 | -12.448968 | -17.960024 | -15.051515 |
| **I-16-66** | -10.728054 | -18.456215 | -14.635712 | -16.555471 |
| **I-16-67** | -19.175505 | -13.20958 | -15.045084 | -15.988873 |
| **I-16-68** | -10.077485 | -11.31314 | -17.108561 | -16.092836 |
| **I-16-69** | -11.720408 | -16.548553 | -15.130264 | -15.417325 |
| **II-23-1** | -18.13312 | -8.925177 | -14.796571 | -11.896244 |
| **II-23-2** | -8.103283 | -11.388297 | -14.945473 | -15.530393 |
| **II-23-3** | -7.937141 | -10.500764 | -15.2622 | -16.405996 |
| **II-23-4** | -11.879715 | -16.306152 | -13.668042 | -10.966539 |
| **II-23-5** | -11.126485 | -11.915153 | -16.085789 | -16.859276 |
| **II-23-6** | -8.768424 | -13.930193 | -14.375991 | -15.705371 |
| **II-23-7** | -10.943602 | -9.621112 | -20.00864 | -9.436154 |
| **II-23-8** | -11.089791 | -18.091106 | -16.096254 | -17.320938 |
| **II-23-9** | -9.167149 | -9.61865 | -12.401745 | -9.903246 |
| **II-23-10** | -10.283531 | -11.67997 | -16.057425 | -15.815566 |
| **II-23-11** | -9.514391 | -14.322066 | -16.250631 | -11.885719 |
| **II-23-12** | -10.421677 | -19.328688 | -16.040342 | -9.466513 |
| **II-23-13** | -9.709284 | -14.040309 | -20.559925 | -11.366633 |
| **II-23-14** | -11.139247 | -10.944977 | -17.87974 | -16.836645 |
| **II-23-15** | -12.914293 | -12.19486 | -22.486687 | -14.202309 |
| **II-23-16** | -11.925481 | -15.268022 | -12.710693 | -9.087912 |
| **II-23-17** | -10.850145 | -10.472358 | -20.752846 | -14.103048 |
| **II-23-18** | -22.447651 | -16.907763 | -15.611929 | -11.345897 |

| | | | | |
|---|---|---|---|---|
| **II-23-19** | -9.332839 | -18.882467 | -14.330648 | -21.789093 |
| **II-23-20** | -11.417106 | -16.571245 | -17.301771 | -22.711149 |
| **II-23-21** | -9.025667 | -12.62611 | -16.372021 | -10.374676 |
| **II-23-22** | -11.292005 | -10.279733 | -14.794174 | -11.837326 |
| **II-23-23** | -9.305825 | -16.235554 | -17.069918 | -13.755506 |
| **II-23-24** | -11.317044 | -16.275534 | -16.834709 | -12.981842 |
| **II-23-25** | -10.897168 | -14.130476 | -19.289455 | -9.398773 |
| **II-23-26** | -27.407663 | -13.64634 | -20.771881 | -24.69978 |
| **II-23-27** | -16.374453 | -8.164698 | -16.261938 | -12.366136 |
| **II-23-28** | -11.417291 | -15.00288 | -15.45627 | -13.073456 |
| **II-23-29** | -10.93737 | -13.817361 | -17.436802 | -19.080004 |
| **II-23-30** | -11.012688 | -7.331734 | -9.799094 | -23.845282 |
| **II-23-31** | -7.835935 | -13.926081 | -16.709721 | -10.513346 |
| **II-23-32** | -12.100845 | -17.183746 | -13.43026 | -12.56481 |
| **II-23-33** | -17.870441 | -13.432671 | -11.943342 | -11.335474 |
| **II-23-34** | -10.894544 | -13.472165 | -15.928277 | -16.163012 |
| **II-23-35** | -11.138191 | -15.740471 | -16.384739 | -6.0232 |
| **II-23-36** | -7.940819 | -12.597077 | -15.41627421 | -11.739987 |
| **II-23-37** | -12.016928 | -14.400308 | -15.473348 | -14.714718 |
| **II-23-38** | -20.142469 | -12.298185 | -15.22328 | -12.049968 |
| **II-23-39** | -10.589848 | -15.954745 | -17.283924 | -18.232361 |

**Table S4**. The structure, free energy of binding, pharmacokinetic and physicochemical properties of the ten best Ami1 inhibitor candidates.

| Compounds | Structures | Docking score (kcal/mol) | Purchasable | SwissADME | | | | |
|---|---|---|---|---|---|---|---|---|
| | | | | Drug likeness (violations) | | | Bioavailability (Score) | Lead likeness |
| | | | | Lipinski | Veber | Muegge | | |
| ZINC4610689 | 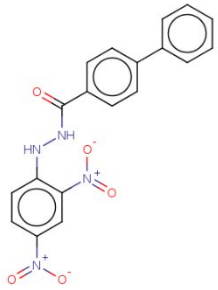 | -7.8 | Available | 0 | 0 | 0 | 0.55 | No |
| ZINC190183 | 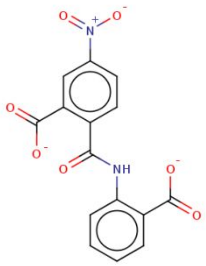 | -7.7 | Available | 0 | 1 | 1 | 0.11 | Yes |
| ZINC2496520 | 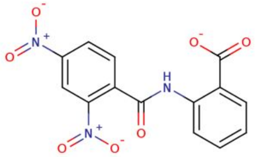 | -7.7 | Available | 0 | 1 | 1 | 0.11 | Yes |

| | | | | | | | | |
|---|---|---|---|---|---|---|---|---|
| ZINC3076022 | 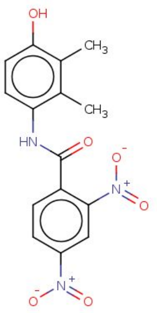 | -7.6 | Available | 0 | 1 | 0 | 0.55 | Yes |
| ZINC5413426 | 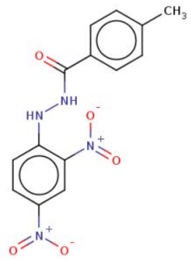 | -7.4 | Available | 0 | 0 | 0 | 0.55 | No |
| ZINC05003646 | 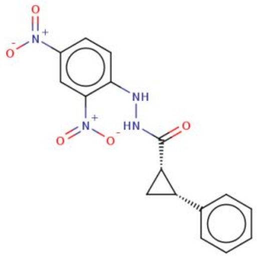 | -7.4 | Available | 0 | 0 | 0 | 0.55 | Yes |
| ZINC5344413 | 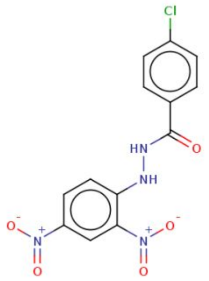 | -7.3 | Available | 0 | 0 | 0 | 0.55 | No |

| | | | | | | | | |
|---|---|---|---|---|---|---|---|---|
| ZINC5513342 | 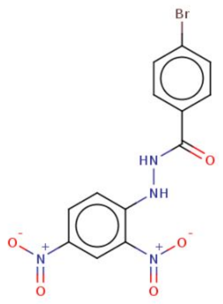 | -7.3 | Available | 0 | 0 | 0 | 0.55 | No |
| ZINC5569515 | 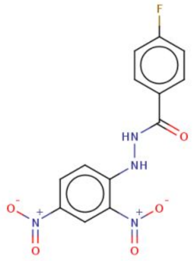 | -7.3 | Available | 0 | 0 | 0 | 0.55 | No |
| ZINC4992193 | 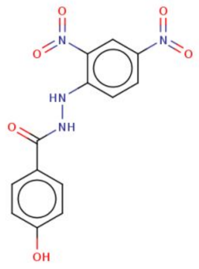 | -7.2 | Available | 0 | 1 | 1 | 0.55 | Yes |

**Table S5.** Crystallographic data

| Data colection statics | Ami1-hit 5 complex |
|---|---|
| Wavelength (Å) | 0.97918 |
| Space group | I $2_1$ $2_1$ $2_1$ |
| Unit cell dimensions<br>a, b, c (Å)<br>α, β, γ (°) | <br>37.35, 103.96, 130.14<br>90, 90, 90 |
| Temperature (K) | 100 |
| Resolution range (Å) | 40.04–1.45 (1.47–1.45) |
| Unique reflections | 44854 (2182) |
| Completeness (%) | 99 (98.2) |
| Multiplicity | 5.3 (5.5) |
| $R_{merge}$ | 0.056 (0.404) |
| $R_{pim}$ | 0.026 (0.184) |
| CC1/2 | 0.999 (0.907) |
| <I/σ (I)> | 16.6 (4) |
| **Refinement statistics** | |
| Resolution range (Å) | 40–1.45 |
| Rwork/Rfree | 0.1798/0.2001 |
| **No. atoms** | |
| Protein | 1580 |
| Water | 160 |
| Ligand | 18 |
| **Root-Mean-Square Deviations** | |
| Bond length (Å) | 0.006 |
| Bond angles (deg) | 0.89 |
| **Ramachandran**<br>Favored/ouliers (%)<br>Residues in the AU | <br>96.67/0<br>212 |
| **Average B-factor**<br>Macromolecules<br>Ligand<br>Solvent | <br>15.07<br>16.20<br>23.14 |
| **PDB code** | 9CUN |

*Values between parentheses correspond to the highest resolution shell.

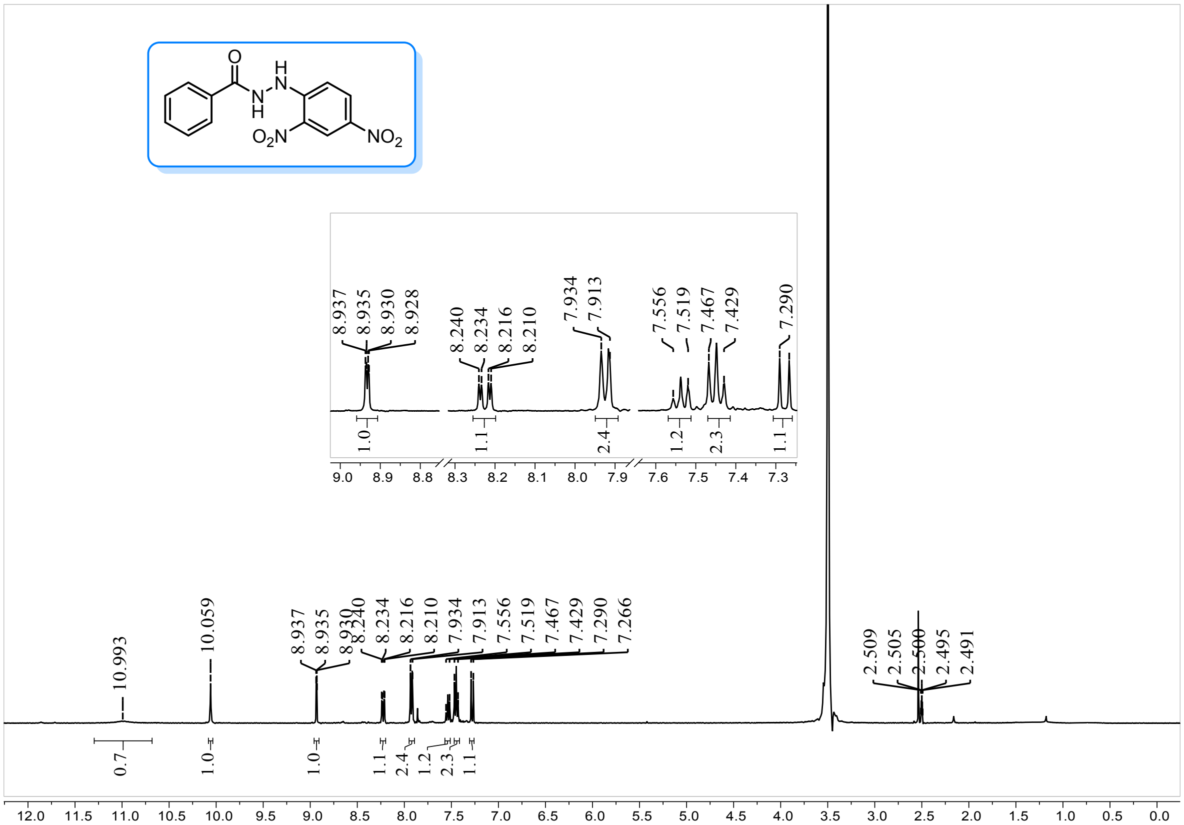


**Spectrum 1**. $^{1}$H-NMR of *N*'-(2,4-dinitrophenyl)benzohydrazide **(1a).**

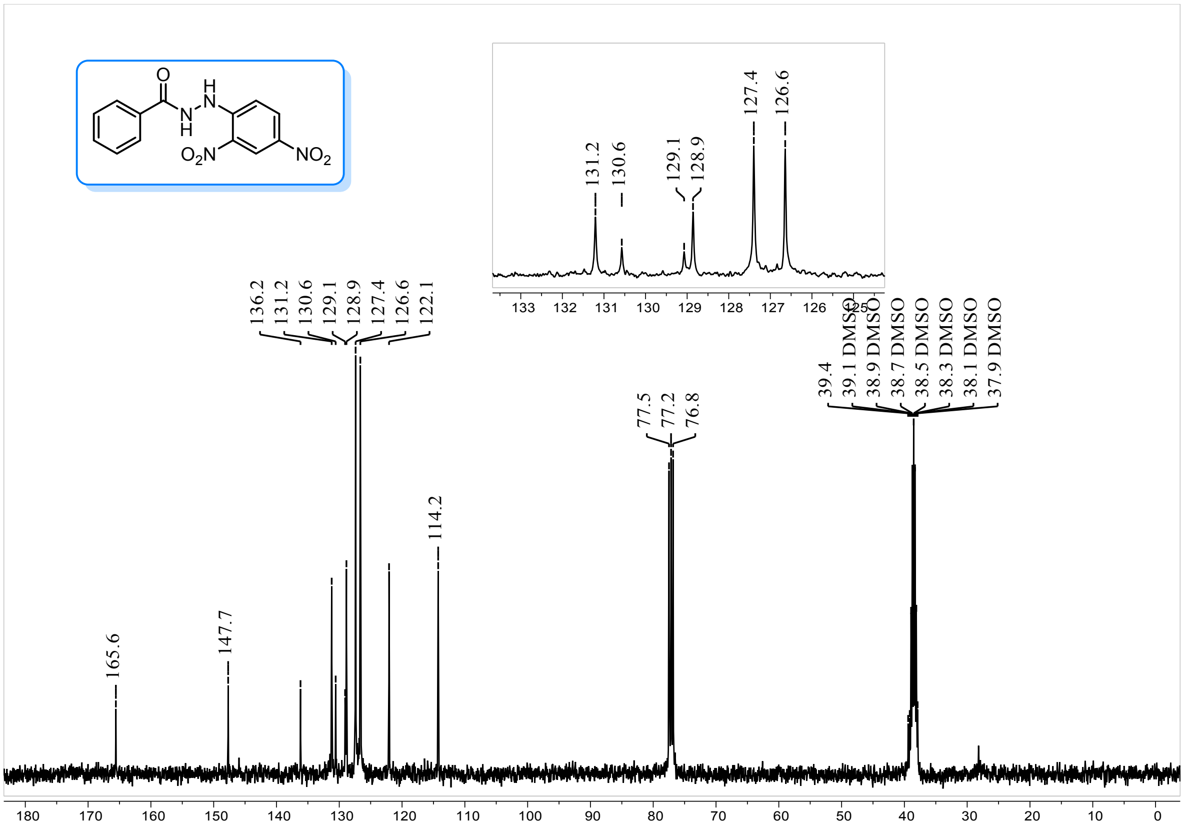


**Spectrum 2**. $^{13}C$-NMR of *N'*-(2,4-dinitrophenyl)benzohydrazide **(1a).**

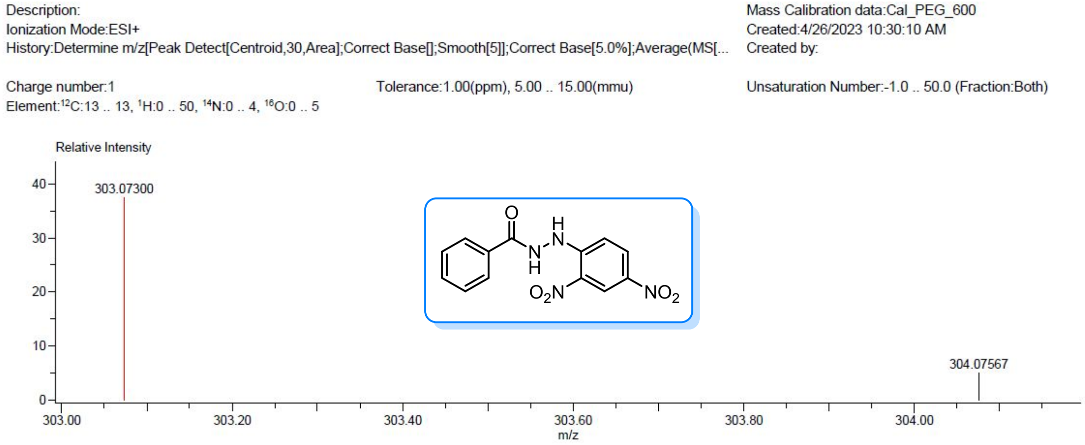


| Mass | Intensity | Calc. Mass | Mass Difference (mmu) | Mass Difference (ppm) | Possible Formula | Unsaturation Number |
|---|---|---|---|---|---|---|
| 303.07300 | 11196.52 | 303.07294 | 0.06 | 0.20 | $^{12}C_{13}{}^{1}H_{11}{}^{14}N_{4}{}^{16}O_{5}$ | 10.5 |

**Spectrum 3.** HRMS of *N'*-(2,4-dinitrophenyl)benzohydrazide **(1a).**

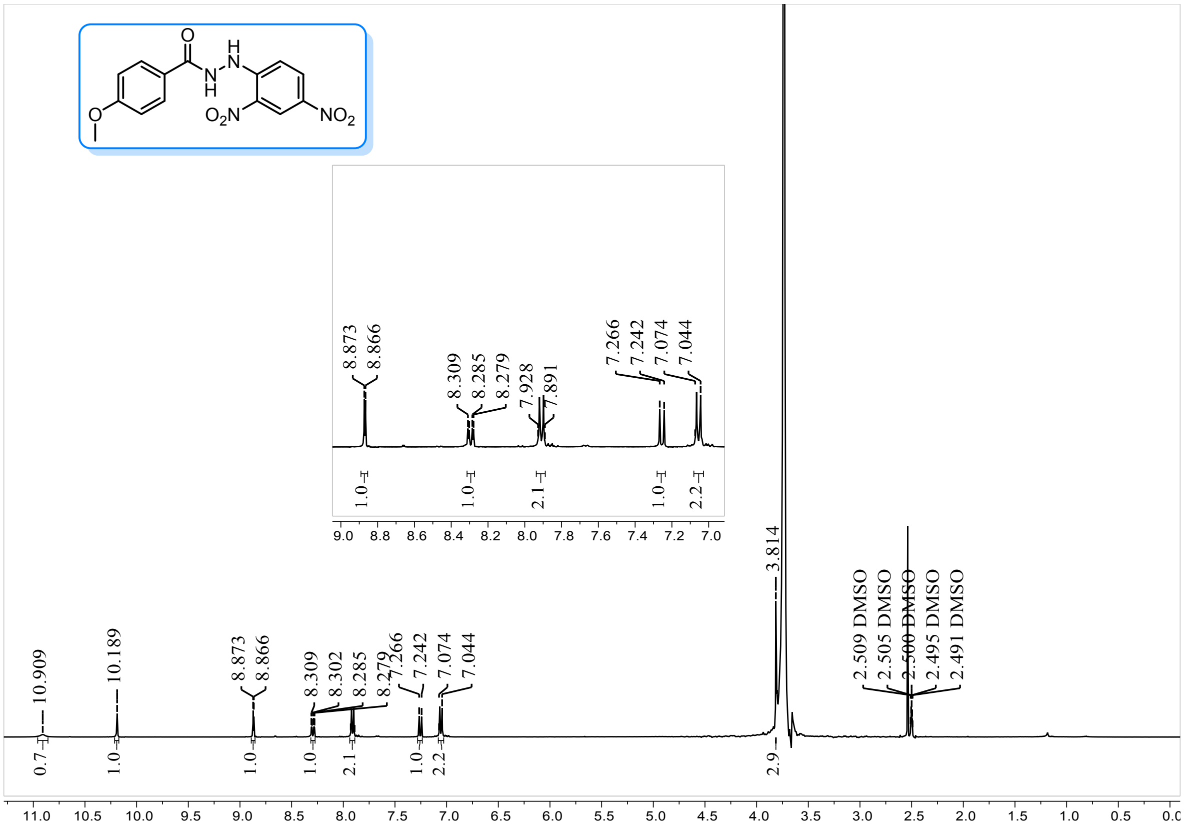


**Spectrum 4**. $^1$H-NMR of *N'*-(2,4-dinitrophenyl)-4-methoxybenzohydrazide (**1b**).

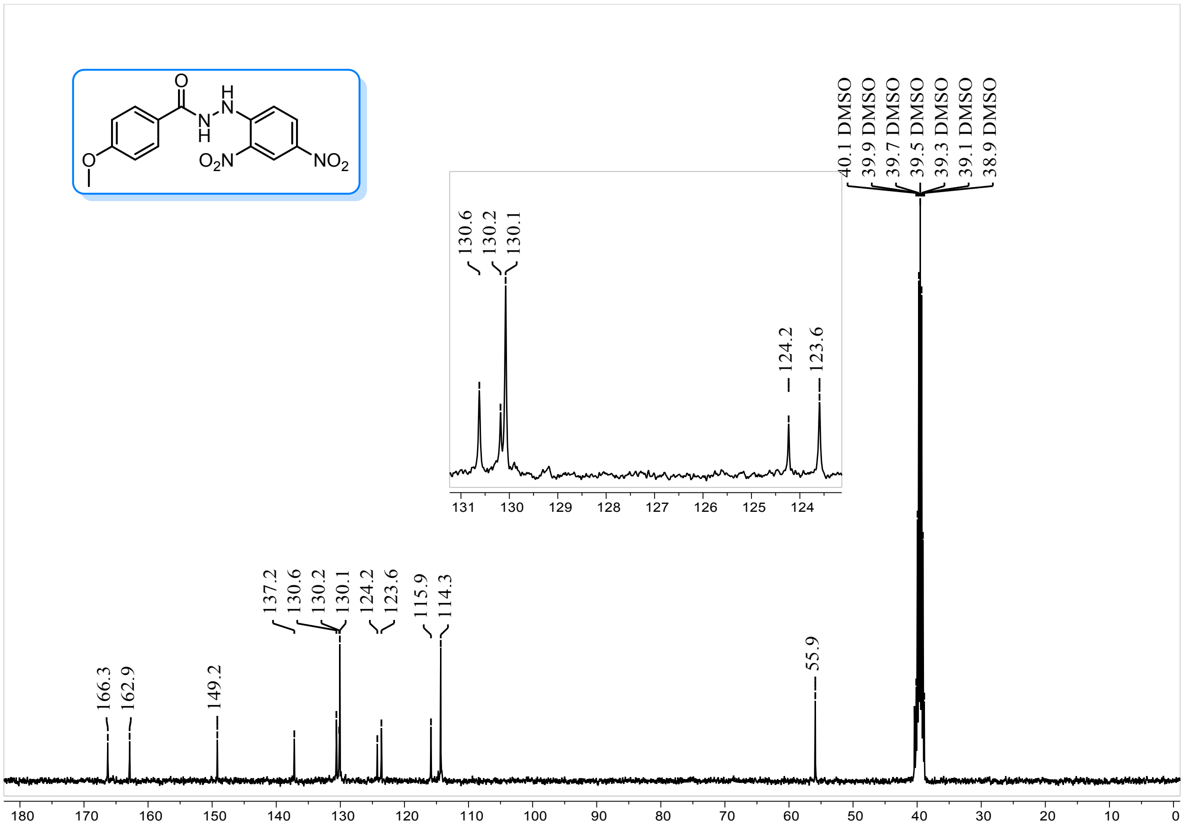


**Spectrum 5**. $^{13}$C-NMR of *N*'-(2,4-dinitrophenyl)-4-methoxybenzohydrazide (**1b**).

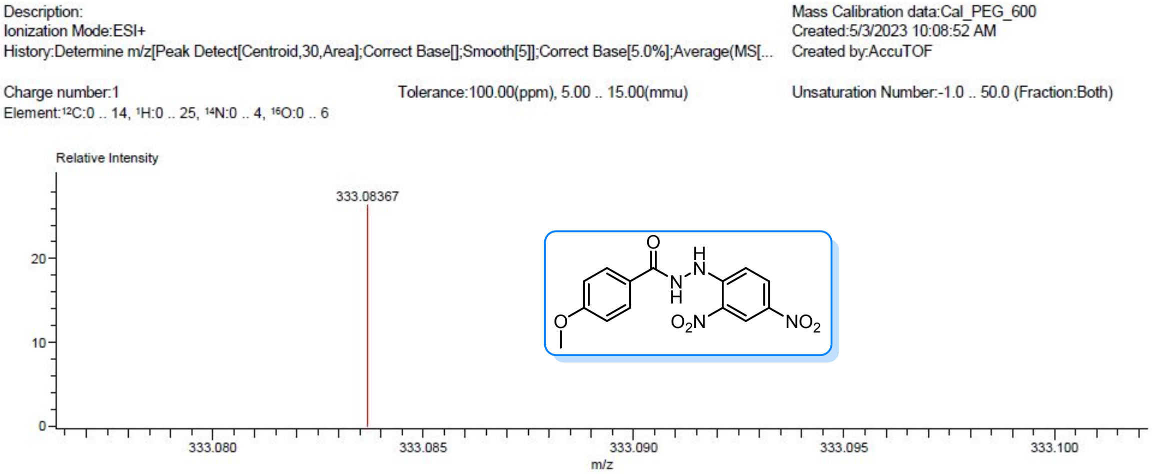


| Mass | Intensity | Calc. Mass | Mass Difference (mmu) | Mass Difference (ppm) | Possible Formula | Unsaturation Number |
|---|---|---|---|---|---|---|
| 333.08367 | 3659.12 | 333.08351 | 0.17 | 0.50 | $^{12}C_{14}{}^{1}H_{13}{}^{14}N_4{}^{16}O_6$ | 10.5 |

**Spectrum 6**. HRMS of *N*'-(2,4-dinitrophenyl)-4-methoxybenzohydrazide (**1b**).

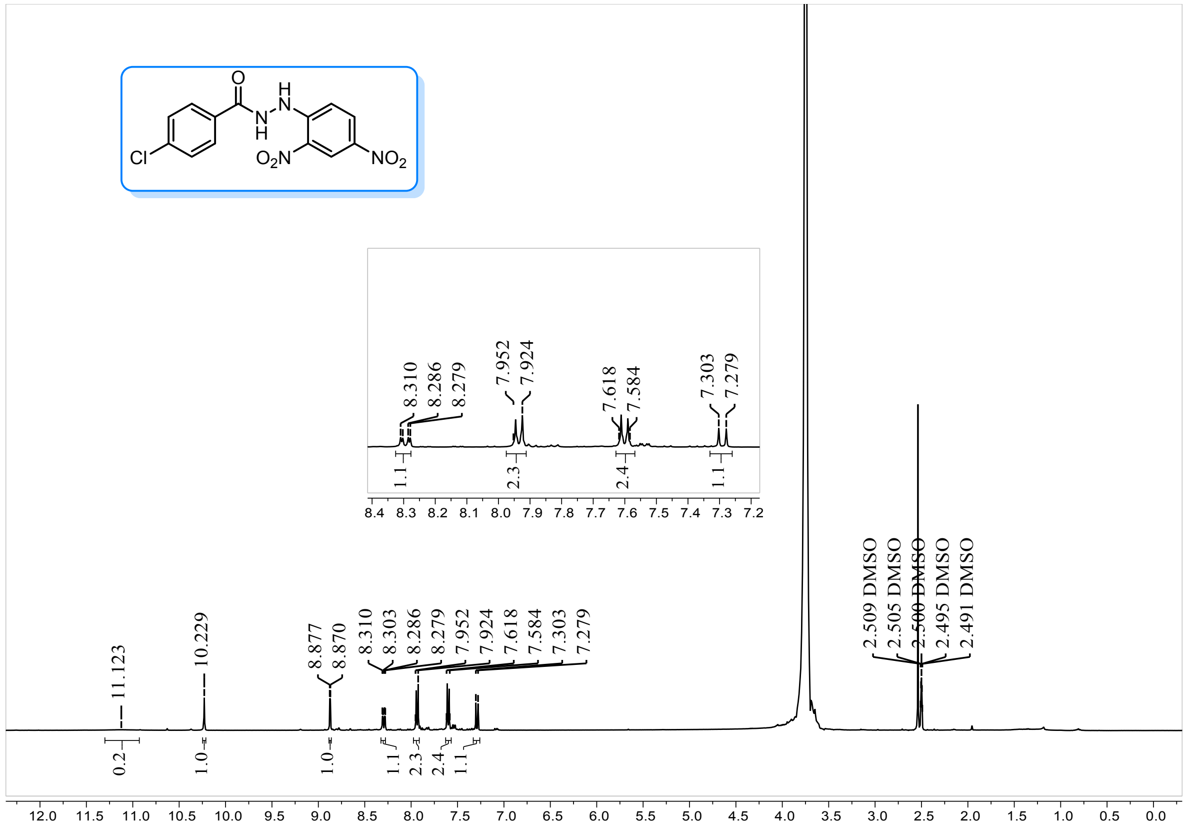

**Spectrum 7**. $^1$H-NMR of 4-chloro-*N'*-(2,4-dinitrophenyl)benzohydrazide (**1c**).

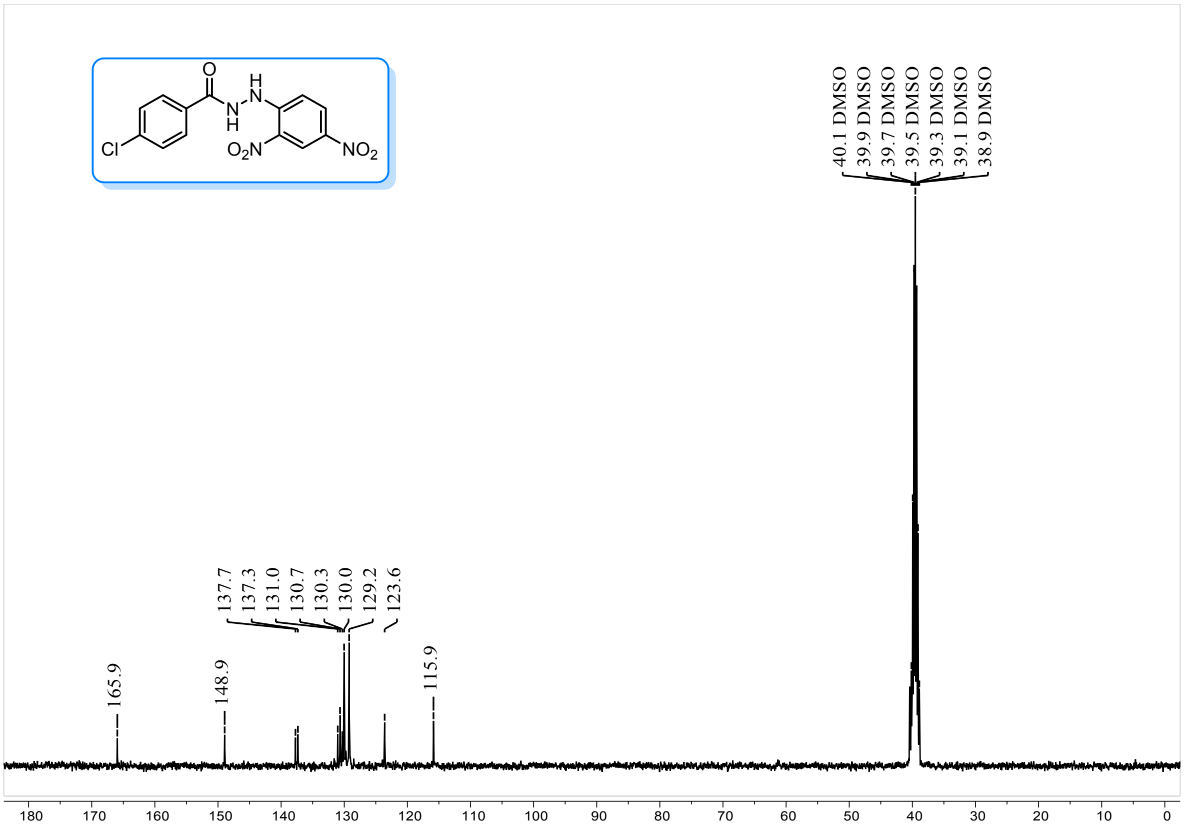


**Spectrum 8**. $^{13}$C-NMR of 4-chloro-*N*'-(2,4-dinitrophenyl)benzohydrazide (**1c**).

Description:
Ionization Mode:ESI+
History:Determine m/z[Peak Detect[Centroid,30,Area];Correct Base[];Smooth[5]];Correct Base[5.0%];Average(MS[...

Mass Calibration data:Cal_PEG_600
Created:6/2/2023 10:32:01 AM
Created by:

Charge number:1
Tolerance:50.00(ppm), 5.00 .. 15.00(mmu)
Unsaturation Number:-1.0 .. 60.0 (Fraction:Both)
Element:$^{12}$C:0 .. 13, $^{1}$H:0 .. 50, $^{35}$Cl:0 .. 1, $^{14}$N:0 .. 4, $^{16}$O:0 .. 5, $^{32}$S:0 .. 0

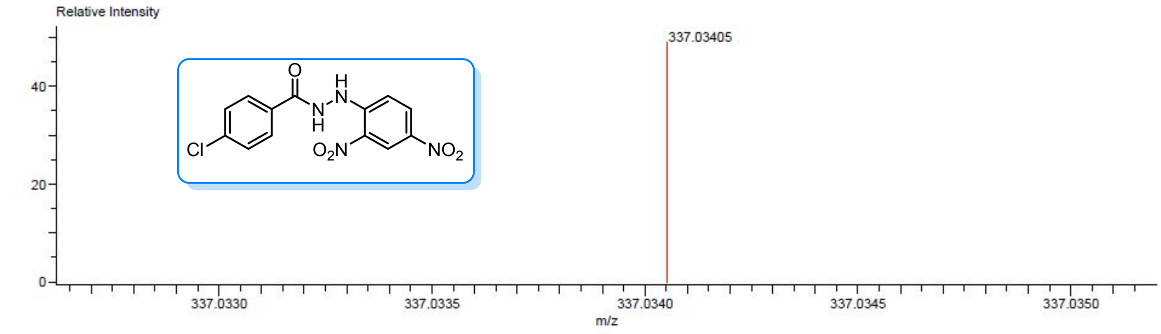


| Mass | Intensity | Calc. Mass | Mass Difference (mmu) | Mass Difference (ppm) | Possible Formula | Unsaturation Number |
|---|---|---|---|---|---|---|
| 337.03405 | 17139.68 | 337.03397 | 0.08 | 0.24 | $^{12}C_{13}{}^{1}H_{10}{}^{35}Cl_{1}{}^{14}N_{4}{}^{16}O_{5}$ | 10.5 |

**Spectrum 9**. HRMS of 4-chloro-*N*'-(2,4-dinitrophenyl)benzohydrazide (**1c**).

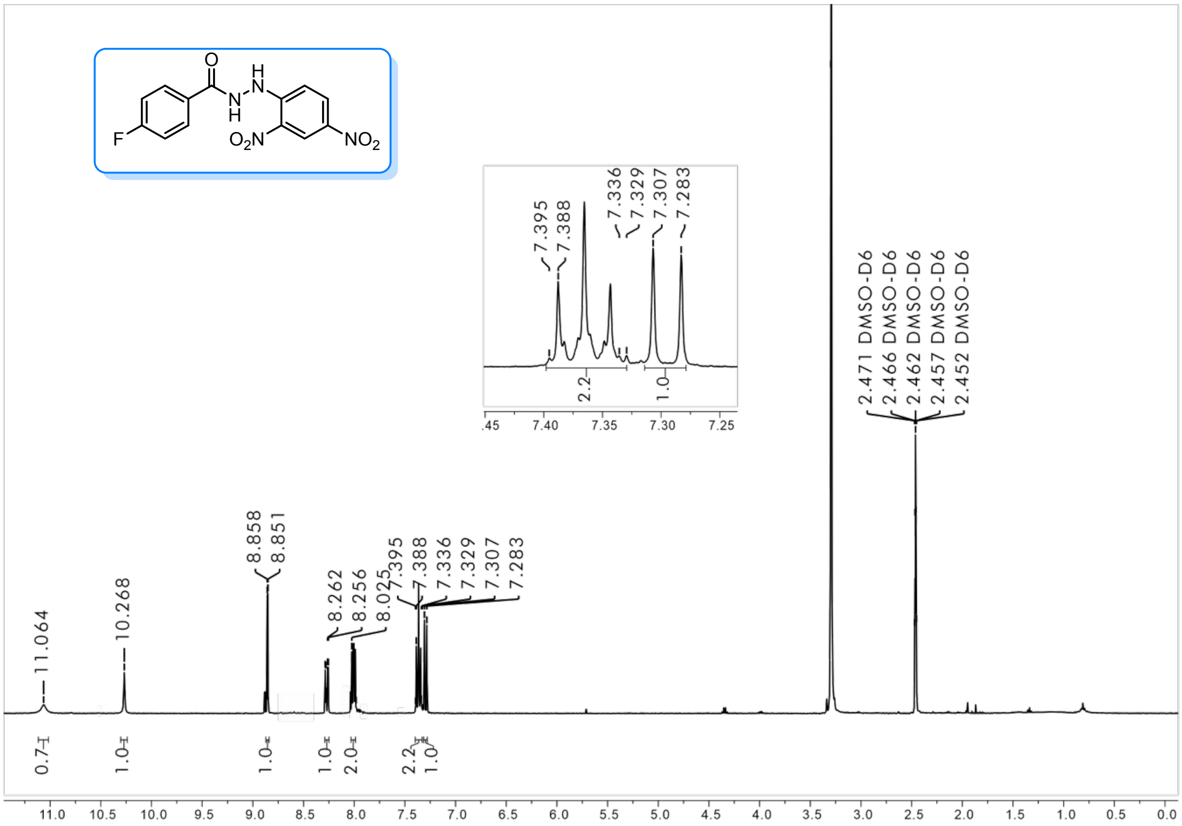


**Spectrum 10**. $^{1}$H-NMR of *N*'-(2,4-dinitrophenyl)-4-fluorobenzohydrazide (**1d**).

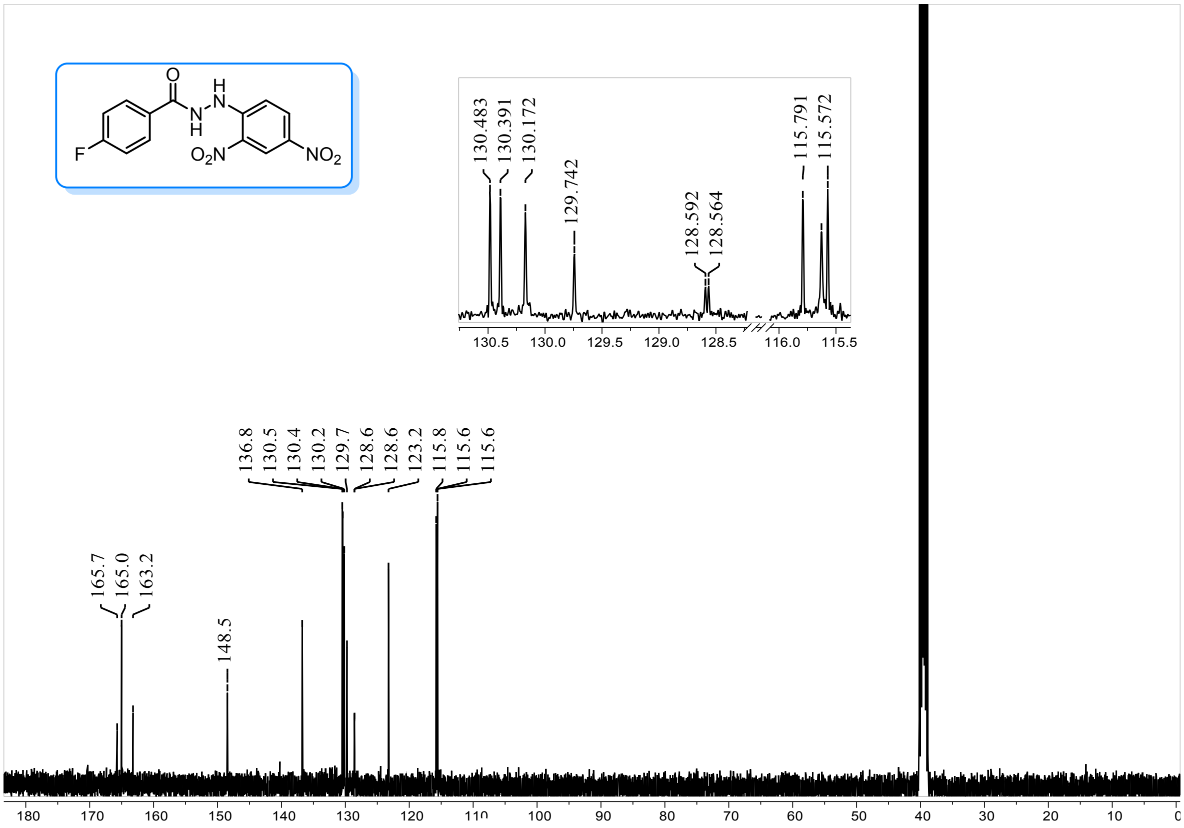


**Spectrum 11**. $^{13}$C-NMR of *N'*-(2,4-dinitrophenyl)-4-fluorobenzohydrazide (**1d**).

Description:
Ionization Mode:ESI+
History:Determine m/z[Peak Detect[Centroid,30,Area];Correct Base[];Smooth[5]];Correct Base[5.0%];Average(MS[...

Mass Calibration data:Cal_PEG_600
Created:6/1/2023 10:41:49 AM
Created by:

Charge number:1
Tolerance:5.00(ppm), 5.00 .. 15.00(mmu)
Unsaturation Number:-1.0 .. 60.0 (Fraction:Both)
Element:$^{12}$C:0 .. 13, $^{1}$H:0 .. 28, $^{35}$Cl:0 .. 0, $^{19}$F:0 .. 1, $^{14}$N:0 .. 4, $^{16}$O:0 .. 5, $^{32}$S:0 .. 0

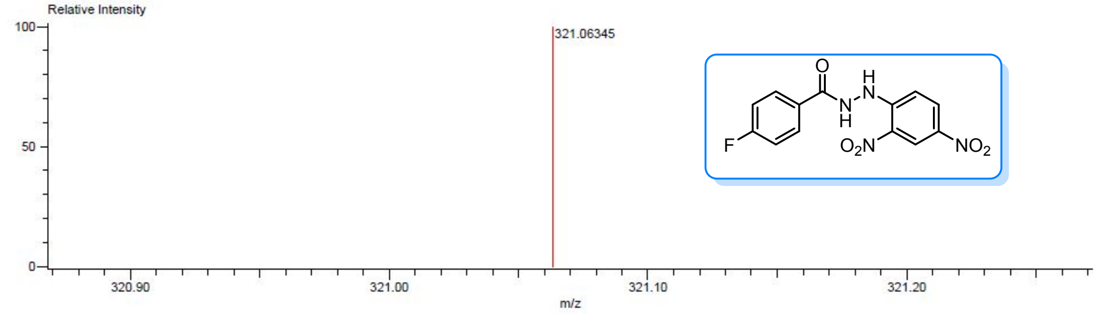


| Mass | Intensity | Calc. Mass | Mass Difference (mmu) | Mass Difference (ppm) | Possible Formula | Unsaturation Number |
|---|---|---|---|---|---|---|
| 321.06345 | 35265.63 | 321.06352 | -0.07 | -0.21 | $^{12}C_{13}{}^{1}H_{10}{}^{19}F_{1}{}^{14}N_{4}{}^{16}O_{5}$ | 10.5 |

**Spectrum 12**. HRMS of *N'*-(2,4-dinitrophenyl)-4-fluorobenzohydrazide (**1d**).

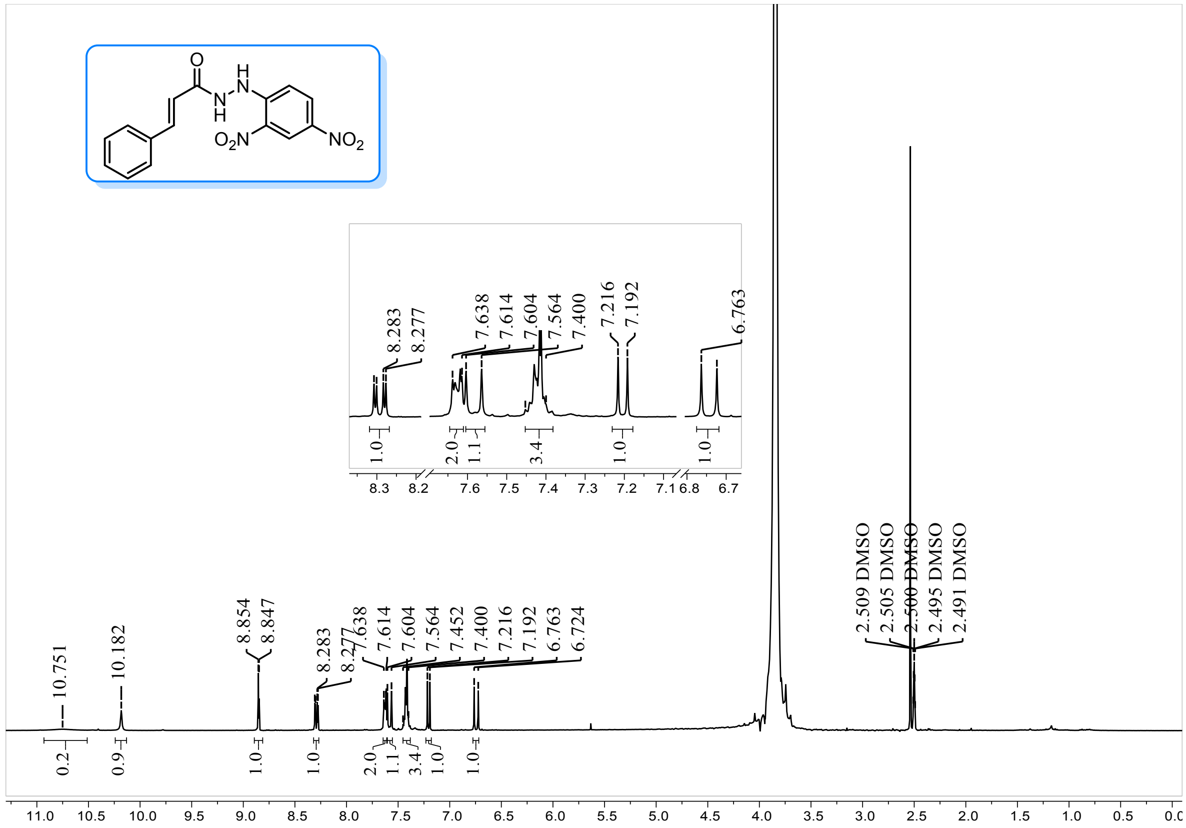


**Spectrum 13**. $^{1}$H-NMR of *N'*-(2,4-dinitrophenyl)cinnamohydrazide (**1e**).

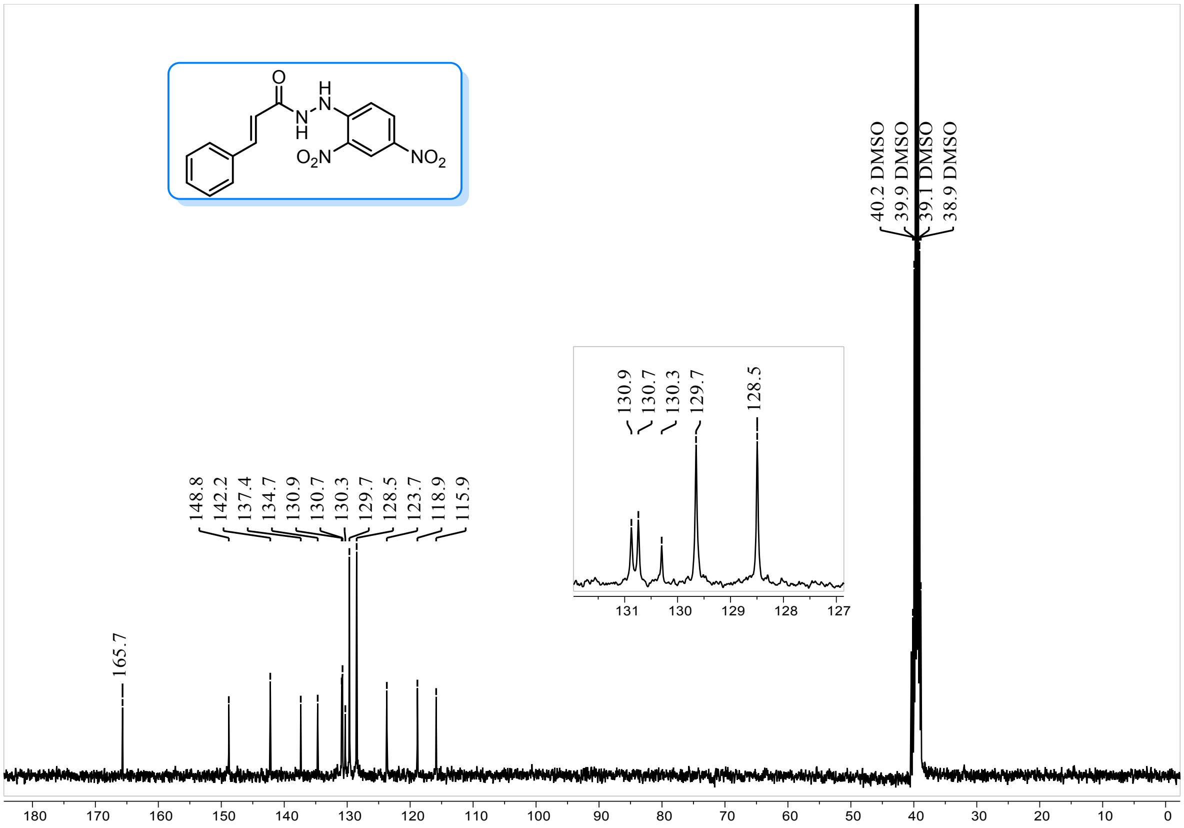


**Spectrum 14**. $^{13}$C-NMR of *N'*-(2,4-dinitrophenyl)cinnamohydrazide (**1e**).

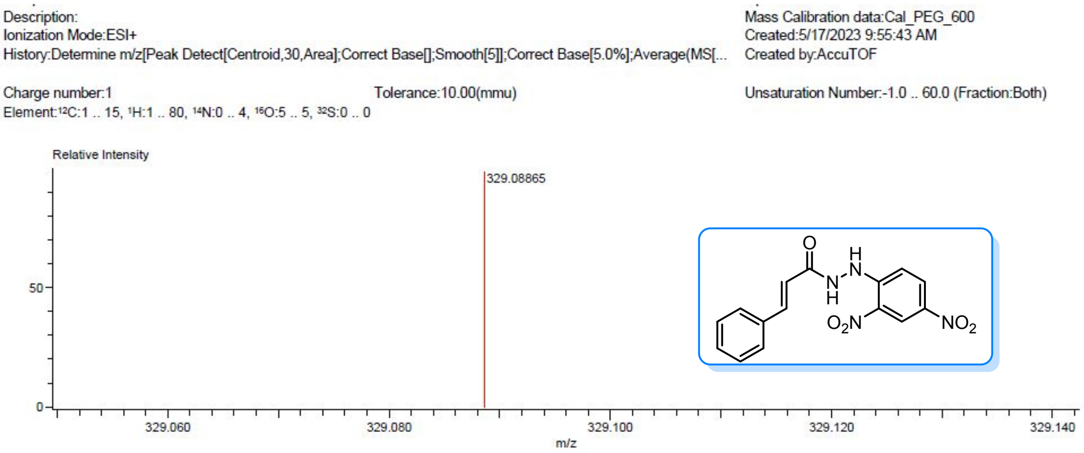


| Mass | Intensity | Calc. Mass | Mass Difference (mmu) | Mass Difference (ppm) | Possible Formula | Unsaturation Number |
|---|---|---|---|---|---|---|
| 329.08865 | 66559.02 | 329.08859 | 0.06 | 0.18 | $^{12}C_{15}{}^{1}H_{13}{}^{14}N_4{}^{16}O_5$ | 11.5 |

**Spectrum 15**. HRMS of *N'*-(2,4-dinitrophenyl)cinnamohydrazide (**1e**).

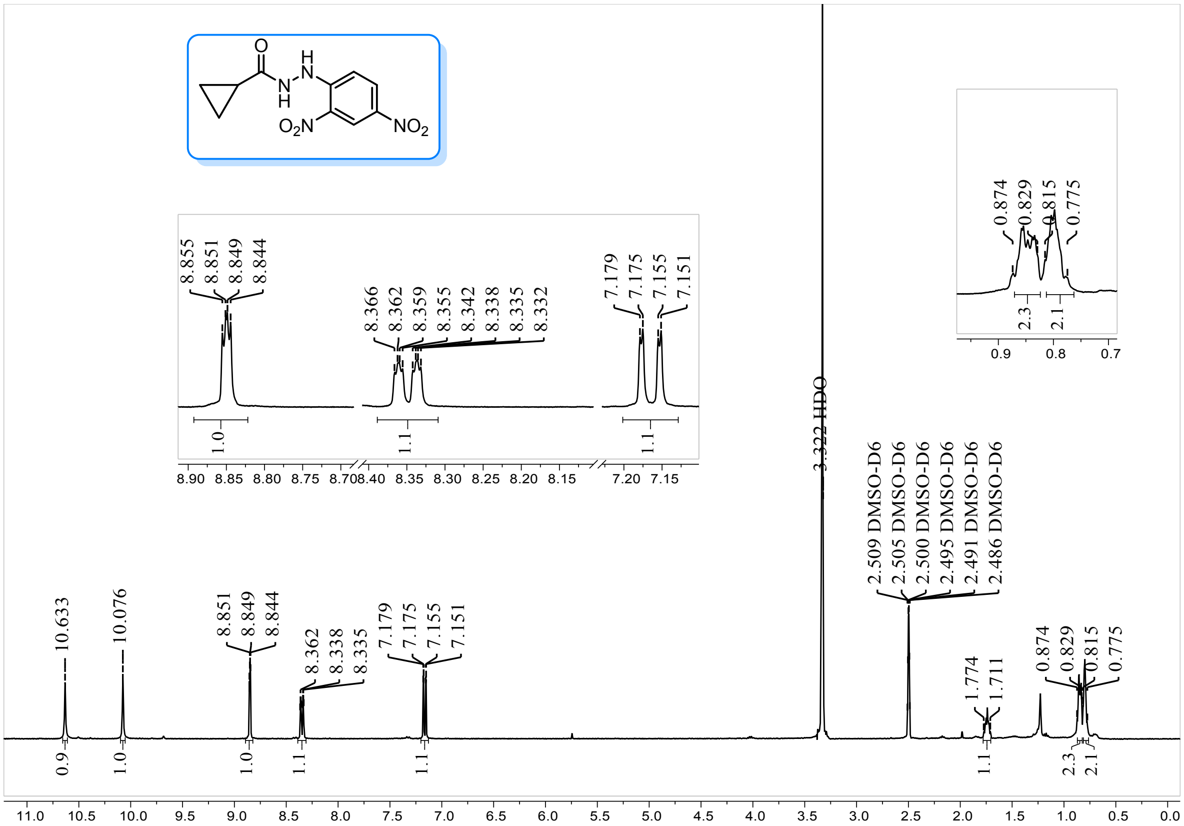


**Spectrum 16**. $^{1}$H-NMR of *N'*-(2,4-dinitrophenyl)cyclopropanecarbohydrazide **(1f).**

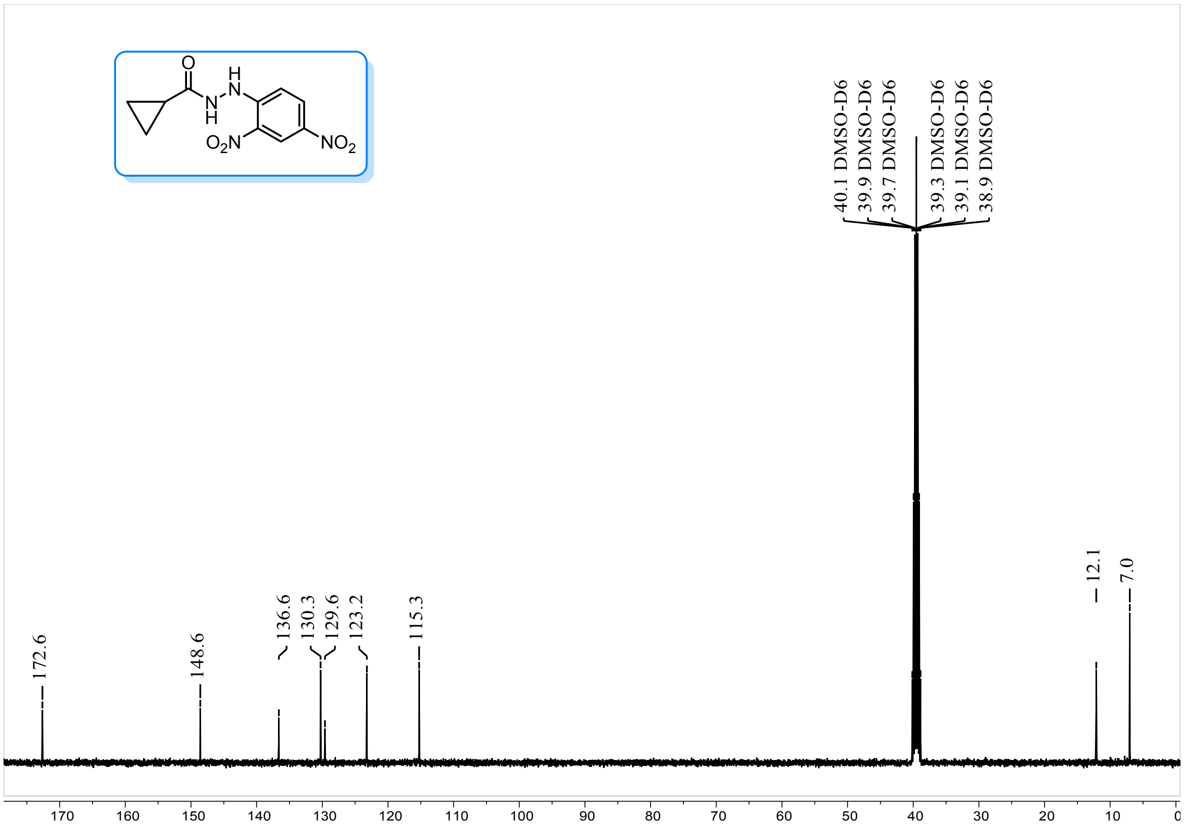


**Spectrum 17**. $^{13}$C-NMR of *N'*-(2,4-dinitrophenyl)cyclopropanecarbohydrazide **(1f).**

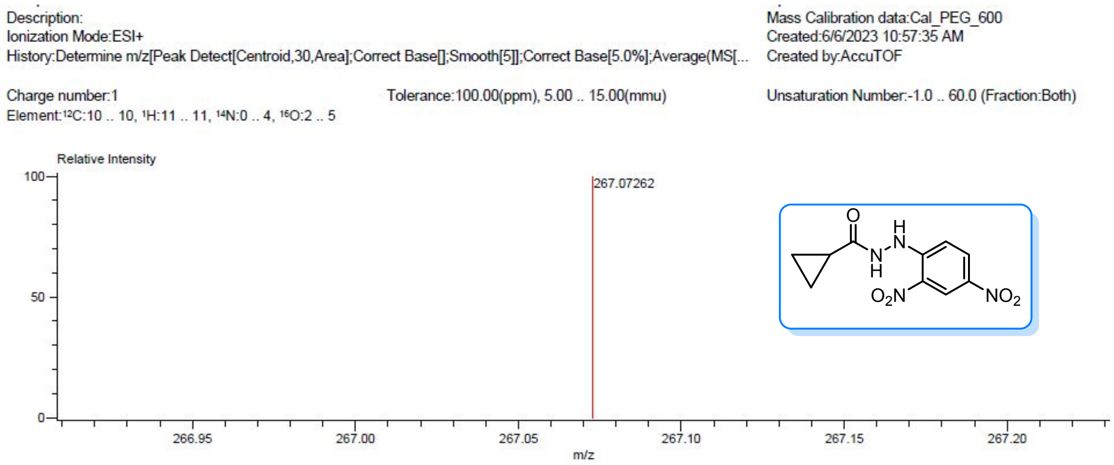


| Mass | Intensity | Calc. Mass | Mass Difference (mmu) | Mass Difference (ppm) | Possible Formula | Unsaturation Number |
|---|---|---|---|---|---|---|
| 267.07262 | 828456.31 | 267.07294 | -0.33 | -1.23 | $^{12}C_{10}{}^{1}H_{11}{}^{14}N_4{}^{16}O_5$ | 7.5 |

**Spectrum 18**. HRMS of *N'*-(2,4-dinitrophenyl)cyclopropanecarbohydrazide **(1f).**

**REFERENCES**


(1) Küssau, T.; Van Wyk, N.; Johansen, M. D.; Alsarraf, H. M. A. B.; Neyret, A.; Hamela, C.; Sørensen, K. K.; Thygesen, M. B.; Beauvineau, C.; Kremer, L.; Blaise, M. Functional Characterization of the N-Acetylmuramyl-l-Alanine Amidase, Ami1, from Mycobacterium Abscessus. *Cells* **2020**, *9* (11). https://doi.org/10.3390/cells9112410.

(2) Wang, W. J.; Cheng, W.; Luo, M.; Yan, Q.; Yu, H. M.; Li, Q.; Cao, D. D.; Huang, S.; Xu, A.; Mariuzza, R. A.; Chen, Y.; Zhou, C. Z. Activity Augmentation of Amphioxus Peptidoglycan Recognition Protein Bbtpgrp3 via Fusion with a Chitin Binding Domain. *PLoS One* **2015**, *10* (10), 1–15. https://doi.org/10.1371/journal.pone.0140953.

(3) Carrasco-López, C.; Rojas-Altuve, A.; Zhang, W.; Hesek, D.; Lee, M.; Barbe, S.; André, I.; Ferrer, P.; Silva-Martin, N.; Castro, G. R.; Martínez-Ripoll, M.; Mobashery, S.; Hermoso, J. A. Crystal Structures of Bacterial Peptidoglycan Amidase AmpD and an Unprecedented Activation Mechanism. *Journal of Biological Chemistry* **2011**, *286* (36), 31714–31722. https://doi.org/10.1074/jbc.M111.264366.

(4) Prigozhin, D. M.; Mavrici, D.; Huizar, J. P.; Vansell, H. J.; Alber, T. Structural and Biochemical Analyses of Mycobacterium Tuberculosis N-Acetylmuramyl-L-Alanine Amidase Rv3717 Point to a Role in Peptidoglycan Fragment Recycling. *Journal of Biological Chemistry* **2013**, *288* (44), 31549–31555. https://doi.org/10.1074/jbc.M113.510792.

(5) Kumar, A.; Kumar, S.; Kumar, D.; Mishra, A.; Dewangan, R. P.; Shrivastava, P.; Ramachandran, S.; Taneja, B. The Structure of Rv3717 Reveals a Novel Amidase from Mycobacterium Tuberculosis. *Acta Crystallogr D Biol Crystallogr* **2013**, *69* (12), 2543–2554. https://doi.org/10.1107/S0907444913026371.
